# Removal of the cervical collar from alpine rescue protocols? A biomechanical non-inferiority trial in real-life mountain conditions

**DOI:** 10.1186/s13049-022-01031-3

**Published:** 2022-06-27

**Authors:** Guillaume Grenier, Marc-Antoine Despatis, Karina Lebel, Mathieu Hamel, Camille Martin, Patrick Boissy

**Affiliations:** 1 Faculty of Medicine and Health Sciences, Department of Surgery, Université de Sherbrooke, Sherbrooke, QC Canada; 2 Faculty of Engineering, Department of Electrical and Computing Engineering, Université de Sherbrooke, Sherbrooke, QC Canada; 3 Research Center on Aging, CIUSSS Estrie CHUS, Sherbrooke, QC Canada

## Abstract

**Background:**

Alpine skiing rescues are challenging because of the mountainous environment and risks of cervical spine motion (CSM) induced during victims’ extrications (EXs) and downhill evacuations (DEs). The benefits of applying a cervical collar (CC) over manual in-line stabilization without CC (MILS) in terms of spinal motion restriction during simulated alpine rescues are undocumented. Our hypothesis was that CSM recorded using MILS alone is non-inferior to CSM recorded with a CC according to a 10 degrees margin.

**Methods:**

A total of 32 alpine extrications and 4 downhill evacuations on different slope conditions were performed using a high fidelity mannequin designed with a motion sensors instrumented cervical spine. The primary outcome was the peak extrication 3D excursion angle (Peak 3D θ_EX,_) of the mannequin’s head. The secondary objectives were to describe the time to extrication completion (tEX) and to highlight which extrication manipulation is more likely to induce CSM.

**Results:**

The median Peak 3D θ_EX_ recorded during flat terrain extrications using CC was 10.77° (95% CI 7.31°–16.45°) compared to 13.06° (95% CI 10.20°–30.36°) using MILS, and 16.09° (95% CI 9.07°–37.43°) for CC versus 16.65° (95% CI 13.80°–23.40°) using MILS on a steep slope. Peak 3D θ_EX_ with CC or using MILS during extrications were equivalent according to a 10 degrees non-inferiority hypothesis testing (*p* < 0.05). Time to extrication completion (tEX) was significantly reduced using MILS without CC on a flat terrain with a median duration of 237,3 s (95% CI 197.8 s, 272.2 s) compared to 358.7 s (95% CI 324.1 s, 472.4 s). During downhill evacuations, CSM with and without CC across all terrain conditions were negligible (< 5°). When CC is used; its installation manipulation induces the highest CSM. When EXs are done using MILS without CC, the logroll initiation is the manipulation inducing the highest risk of CSM.

**Conclusion:**

For experienced ski patrollers, the biomechanical benefits of spinal motion restriction provided by CC over MILS during alpine skiing rescues appear to be marginal and CC use negatively affects rescue time.

**Supplementary Information:**

The online version contains supplementary material available at 10.1186/s13049-022-01031-3.

## Introduction

After road traffic accidents and falls, sport accidents are the most frequent cause of traumatic spinal injuries (TSI) [1], with alpine winter sports representing about 20% of these [2–4] and the cervical spine being implicated in about 50% of cases [5]. Recent data suggests the aim of pre-hospital care should be to achieve spinal motion restriction (SMR) rather than full immobilization [6]. SMR may be achieved using manual in-line stabilization (MILS). Orthotic devices may be used as well but are challenged because data indicate that the proposed benefits do not always outweigh the related risks [7–12]. Cervical collars (CC) may lead to serious complications such as pressure ulcers, airway difficulties, increased intracranial pressure, increased imaging and radiation exposure [13–21]; neurologic aggravations in ankylosing spondylitis [22, 23] and elderly patients [24, 25]; and increased mortality in penetrating trauma patients [10, 26, 27]. Moreover, CCs installation requires precious time as well as winter clothing and helmet removal, inducing higher risks of CCs inappropriate use, victim cold exposure, and other hazardous events [28].

The Canadian ski patrol follows the National Institute for Health and Care Excellence guidelines [29]. According to these, ski patrollers should use a CC for rescues unless they can safely clear the C-spine before the victim extrication. Other guidelines include the Wilderness Medical Society [9] which despite its main recommendation in favor of the cervical collar, mentions that in some situations "[the cervical collar] should not be considered necessary if adequate immobilization can be accomplished by other means" which include manual in-line stabilization (MILS) [9]. Kornhall et al. [11] recently published prehospital Norwegian Guidelines emphasizing the limited evidence on CCs efficacy and supporting a selective approach to achieve timely rescues. The First Aid Task Force 2020 recommendations suggests against the use of cervical collars by first aid providers (weak recommendation, very low-quality evidence) and concluded that there is insufficient evidence for or against manual in-line stabilization [30].

Thus, current guidelines regarding CCs use are partly conflicting and rely mostly on weak recommendations based on low-quality evidence making CCs and full immobilization protocols still frequently used [7, 10, 14, 16, 26, 31–33]. More data is needed in real-life alpine skiing rescue situations to strengthen evidence.

Simulation based studies with simulated victims during rescue scenarios and motion capture technology have been used to explore and evaluate best practices for SMR [8, 34]. Various 3D motion capture technologies including optical motion capture, magnetic tracking systems, and inertial movement systems are used to look at the impact of SMR of segmental motion of the head and trunk [35]. Optical systems remain the gold standard but require a clear sightline to markers, making it technically difficult to use in situations with multiple rescuers crowding the victim. Magnetic systems operated under constrained volumes are not ultra-responsive to fast motion. Wearable Inertial measurement units (IMU) offer more flexibility to study SMR under field conditions, have good accuracy for short data recordings but require a sensor to body calibration (IMUs’ record orientation in a global coordinate system that is not anatomically aligned with a specific joint coordinate system) which can affect their accuracy. Simulated victims can’t remain reproduce an unconscious person with no muscle tone and placed in an uncomfortable and dangerous position for long periods of time. As an answer to these issues, a high-fidelity humanoid simulation mannequin with a 4-segment mechanical cervical spine instrumented with motion sensors was used in this study to accurately capture CSM [36].

The aim of this study was to evaluate CSM variations occurring in alpine skiing rescues depending on the use or not of a cervical collar (CC versus MILS) during simulated extrications (EXs) and downhill evacuations (DEs) on real-life ski mountain terrains with a high-fidelity simulation mannequin. Our hypothesis was that according to a 10 degrees margin, cervical spine motions recorded using MILS without a cervical collar is non-inferior to cervical spine motions recorded with a cervical collar during ski rescue.

## Methods

### Design and participants

This was a biomechanical pre-experimental simulation-based study. Data was collected on February 22nd and 23rd 2020 at the Mont-Orford Ski Resort in Quebec, Canada. Six volunteer active ski patrollers of the Quebec’s Eastern Townships Region Canadian ski patrol were recruited to participate in this study. Participants were aged between 30 and 56 years old and had between 9 and 21 years of ski patrol rescue experience. Patrollers of the Canadian ski patrol must succeed a 56 h course including theory as well as on field teaching followed by certification exams. An average minimum of sixteen active days as ski patrollers is afterwards required yearly. No medical training is required. All participants were informed of the risks associated with real-life conditions data collection and provided informed consent before data collection.

### High fidelity mannequin and motion capture system

The high-fidelity mannequin used to collect CSM during alpine rescue simulations was developed by our group based on previous c-spine management best practice research [37–39]. The mannequin measures 175 cm and weighs 82 proportionally distributed kilograms. It has a full humanoid silicone-based shape with an articulated internal skeleton reproducing the physiological range of movements and segments weight of each body segment. The mass and the center mass of each segment are distributed according to published values from cadavers’ studies adjusted for the anthropometrical dimension of the mannequin. The articulated internal skeleton is over molded with silicone with a shoreness close to skin tissue to reproduce bone to soft tissue interface and provide a skin like feel when manipulating the mannequin. The anthropometrical and biomechanical validity of the mannequin was examined informally with extensive field research testing and training (over 300 h of use in different c-spine scenarios) with over 150 first responders (including paramedics, firefighters, ski patrollers) and medical professionals (including orthopedic surgeons, neurosurgeons, anesthesiologists and nurses) that subjectively confirmed the realism of the mannequin cervical motions and the high fidelity of the mannequin when as used a proxy for an unconscious human during c-spine mobilization and transfer in the field.

**Fig. 1:**
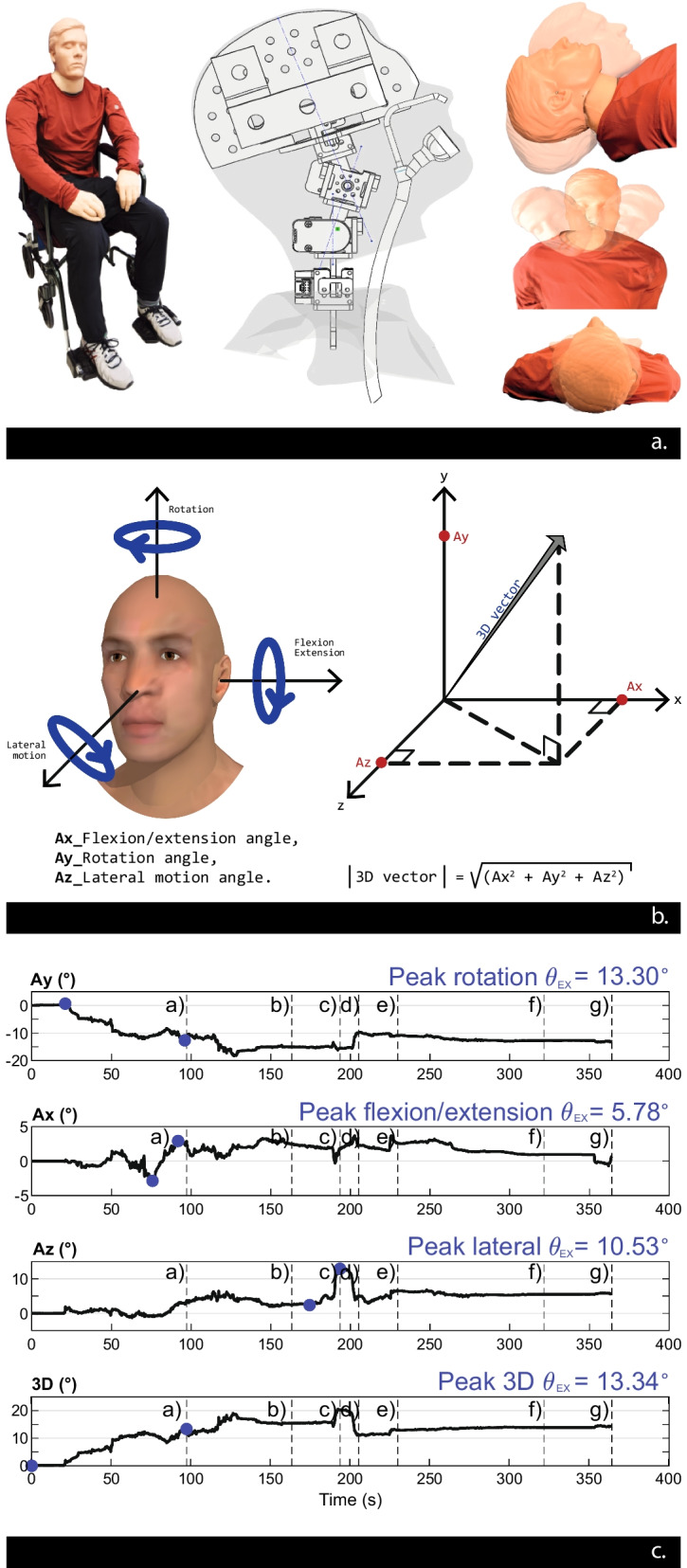
High-fidelity mannequin with integrated cervical spine motion capture system. (**a**) High-fidelity mannequin morphology and integrated motion capture system; (**b**) head motion elementary axes and 3D angle calculation; (**c**) continuous raw data obtained from a single extrication using a cervical collar. Vertical lines mark the extrication’s manipulation divisions (see Fig. 2). θ_EX_: excursion angle

The cervical spine consists of a 4-segment mechanical structure reproducing head motion in all anatomical planes [36] (see Additional files 1 and 2). Rotary optical encoders (sensor that encodes a position and can measure motion as changes in angular position over time) with an accuracy of 0.073 deg (resolution of 5000 counts per revolution over 360 degrees) are positioned in each segment, daisy-chained, and linked to data acquisition board sampling their data at 100 Hz and transmitting it wirelessly to a tablet device for real-time and asynchronous head motion data analysis. Head motion derived from the motion recorded by the linear optical encoders is divided into three elementary axes: (1) flexion/extension; (2) lateral motion, and (3) rotation (see Fig. 1). A 3-dimensional angle is calculated to depict the overall head motion at every moment. The peak variation in time of this 3D angle over a complete extrication process is the extracted primary outcome referred to as the peak extrication 3D excursion angle (Peak 3D θ_EX_). Only the magnitude of this 3D angle is reported as it is considered that its direction has no clinical value. Regarding the secondary outcome, the peak variation of the 3D angle within each manipulation is also extracted and referred to as the peak manipulation 3D excursion angle (Peak 3D θ_M_).

### Data collection: manipulations and terrains

**Fig. 2:**
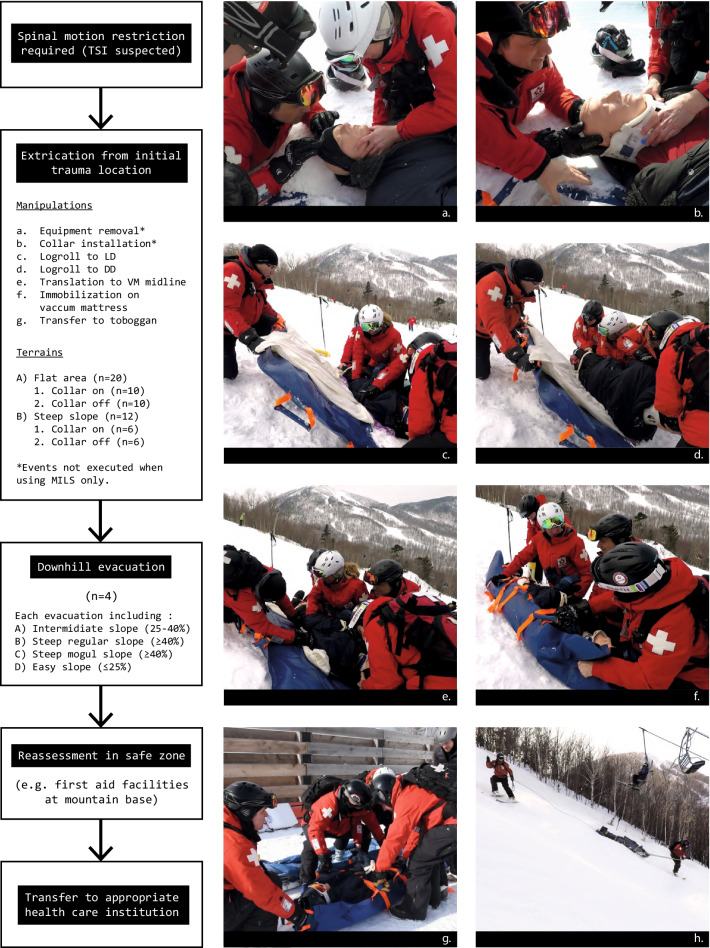
Alpine skiing rescue process and description of terrains. **a** Winter clothes removal and **b** cervical collar installation (flat terrain); **c** preparing logroll to lateral decubitus and **d** preparing logroll back to supine (steep slope); **e** preparing to move to vacuum mattress midline and **f** immobilization on the vacuum mattress (steep slope) (note that head and cervical spine are included in the vacuum mattress immobilization), **g** preparing transfer to toboggan, **h** downhill evacuation (steep mogul slope). LD: lateral decubitus, DD: dorsal decubitus, VM: vacuum mattress, MILS: manual in line stabilization, TSI: traumatic spine injury

Figure 2 summarizes the mountain rescue steps. The first step is the extrication (EX), in which the mannequin is mobilized from its initial trauma location to a rescue toboggan. It is further divided into manipulations (a to g) and culminates with the transfer to the toboggan. The downhill evacuation (DE) phase starts the moment the mannequin is secured in the toboggan and ends when it reaches the base of the mountain.

To understand alpine geography’s impact on the ability to maintain an appropriate cervical alignment, extrications were tested on two different terrains: (A) flat terrain; (B) steep slope (≥ 40% grade). In a similar manner, downhill evacuations were executed on four different inclinations trails, from an easy regular descent < 25% to a ≥ 40% mogul descent. All events were completed an equal number of times with a CC and using MILS (no CC).

### Technique standardization

Simulation conditions (position and dressing of the mannequin, location, start and ending endpoints, equipment used) and the exact protocol (tasks to perform between start and ending endpoints, phase segmentation) executed during each trial were scripted and standardized. At the beginning of every rescue simulation, the mannequin was positioned supine with the head in the neutral position and dorsal spine aligned with the head. It was dressed and geared as a typical alpine skiing victim, including a helmet, ski goggles, and a neck warmer. When using a CC, ski patrollers first removed this equipment to achieve proper CC positioning. When no CC was used, the equipment was kept in place and MILS was applied until the mannequin was transferred to the toboggan. Proper CC fitting to the mannequin and positioning by all patrollers was validated as this is a frequent issue limiting cervical collars effectiveness [28]. Regarding the MILS technique (head squeeze vs trap squeeze) the choice was left to each patroller as some data suggests first responder tend to be more proficient with either the former or later option, most likely depending on their personal preferred technique [38]. Although the lift and slide technique to position the victim into the vacuum mattress might be more effective [37, 40–42], the log roll technique was used in this study considering most first aid providers are familiar with it and have the advantage to require fewer people to be properly executed. A full-body, first-aid-provider vacuum mattress, instead of a long spine board was used because growing evidence supports that it is at least as efficient as the spinal board and has fewer associated complications [7, 43–45]. When the CC was used, it was left in place on the victim throughout the steps of the transfer to the vacuum mattress and toboggan. Prior to the transfer to the toboggan, the vacuum mattress was molded around the mannequin’s shoulders, head, and neck using the vacuum. The CC used was a Laerdal stifneck collar (Canada), the vacuum mattress was a Certec anatomic mattress MFR 100-90 (France) and the toboggan was a Cascade Rescue Company model 100 The Legend (USA).

The ski patrollers had the opportunity to do multiple practice runs with the scripted standardized protocol in each location (flat, slope, and evacuation) and assume lead roles (head stabilization) before the data trials. Data collection was designed so that learning and fatigue bias were lessened while as many repetitions as possible could be executed despite logistic limitations and time constrains. CC use and non-use as well as patrollers’ role were alternated at every repetition. As an example, trial #1 was planned to be with a CC, patroller A stabilizing the head, patroller B at the torso, patroller C at the hips, patroller D at the feet and patroller E bringing the vacuum mattress. Trial #2 was planned to be MILS alone, patroller E stabilizing the head, patroller A at the torso, and so on for all repetitions. Patrollers’ role (A, B, C, D, E) were randomly assigned on the morning of the trials. For practicality and logistics reasons, terrain conditions were not alternated during the data acquisition (data was first gathered on a flat terrain followed by a heavy slope).

### Data analysis

Figure 1 demonstrates an example of raw data collected from a single extrication (EX). The primary outcome extracted from each repetition is the peak extrication 3D excursion angle (Peak 3D θ_EX_). The Peak 3D θ_EX_ is the highest head motion variation, all axis combined, recorded during a whole extrication. Time to extrication completion (tEX) was obtained for each repetition. Regarding secondary outcomes, EXs are subdivided into manipulations (see Fig. 2) and peak manipulation 3D excursion angles (Peak 3D θ_M_) for each of these are extracted to assess which manipulation induces the highest risk of CSM.

Data analysis was conducted using IBM SPSS Statistics Version 26. *P* values < 0.05 were considered significant. No a priori power estimation was performed. Shapiro–Wilk test demonstrated non-normal distribution. Differences in Peak 3D θ_EX_ (primary outcome) recorded between conditions (CC, MILS) during extrications (EXs) and downhill evacuations (DEs) were assessed using Mann–Whitney U non-parametric tests with a 10 degrees non-inferiority hypothesis testing. Since there are no known minimal clinical important difference (MCID) for spinal motion in the context of mobilization and transfer of victim with a potential spine injury, we chose a conservative value of 10 degrees as a proxy for a failed application of SMR. This failure criteria is based on observations from a previous simulation study assessing the effectiveness of different applications of SMR and comparing with sensitivity analysis of head motions recorded with IMUs to performance perception (success, partial success and failure) of lead rescuers and simulated patients during mobilization conditions [46].

## Results

A total of 32 extrications (EXs) were performed. Out of these, 20 were on a flat area and 12 on a 40% grade slope (see Fig. 2). In addition, four downhill evacuations (DEs) were executed. An equal number of repetitions were done with and without a CC for both EXs and DEs.

### Cervical spine motion during extrication

**Fig. 3:**
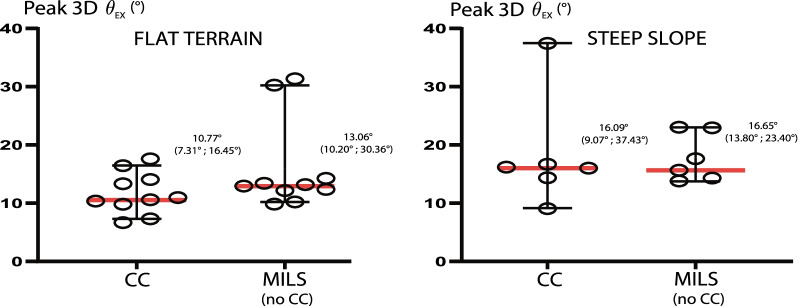
Peak 3D excursion angle during extrication trials on flat terrain and steep slope. Left: Peak extrication 3D excursion angles on a flat terrain depending on the use or not of a cervical collar; Right: Peak extrication 3D excursion angles on a steep slope (> 40% grade) depending on the use or not of a cervical collar. Medians and 95% confidence intervals are represented. Peak 3D θ_EX_: peak extrication 3D excursion angle, CC: cervical collar, MILS: manual in-line stabilization

Figure 3 shows the cervical spine motion (Peak 3D θ_EX_) recorded during each extrication (EX), depending on CC or MILS use, and on terrain conditions. Extrications with CC on a flat terrain induced a median Peak 3D θ_EX_ of 10.77° (95% CI 7.31°–16.45°) compared to a median Peak 3D θ_EX_ of 13.06° (95% CI 10.20°–30.36°) during EXs using MILS without a cervical collar. Further CSM during EXs on a steep slope using a CC reached a median Peak 3D θ_EX_ of 16.09° (95% CI 9.07°–37.43°) whereas on the same terrain using MILS the median Peak 3D θ_EX_ was 16.65° (95% CI 13.80°–23.40°). CSM during EXs with CC and MILS are equivalent according to a 10 degrees non-inferiority hypothesis testing (*p* < 0.05).

### Time to extrication completion

**Fig. 4:**
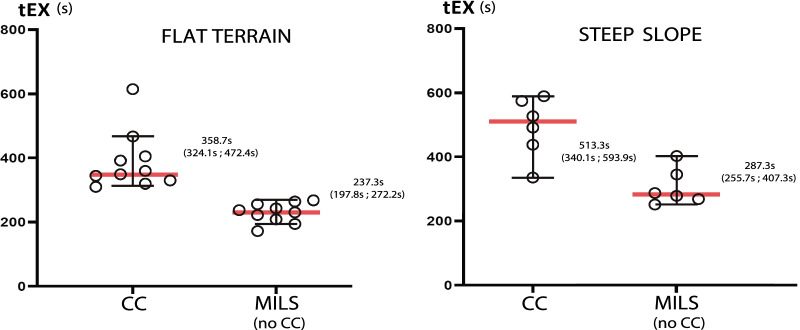
Time to extrication completion depending on the collar use and terrains. Left: Time to extrication completion on a flat terrain depending on the use or not of a cervical collar; Right: Time to extrication completion on a steep slope (> 40% grade) depending on the use or not of a cervical collar. Medians and 95% confidence intervals are represented. tEX: time to extrication completion (seconds), CC: cervical collar, MILS: manual in-line stabilization

Median extrication time (tEX) on flat terrain with a CC was 358,7 s (95% CI 324.1–472.4 s) compared to 237.3 s (95% CI 197.8–272.2 s) when no CC is used (p < 0.05) (see Fig. 4). A similar result was obtained when extrications were performed on a steep slope, the median tEX using CC was 513.3 s (95% CI 340.1–593.9 s) compared to 287,3 s (95% CI 255.7–407.3 s) (p < 0.05).

### Cervical spine motion during a downhill evacuation

**Fig. 5:**
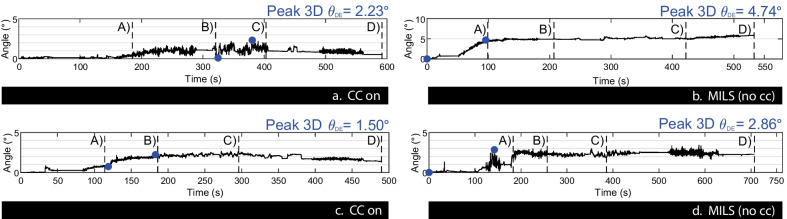
Continuous data collected during downhill evacuations depending on terrains and cervical collar use. (**a**) First downhill evacuation executed with a cervical collar; (**b**) second downhill evacuation executed with manual in-line stabilization; (**c**) Third downhill evacuation executed with a cervical collar; (**d**) fourth downhill evacuation executed with manual in-line stabilization. Vertical lines mark the slope terrain transitions: (A) Intermediate slope (25–40% grade), (B) Steep regular slope (≥ 40% grade), (C) Steep mogul slope (≥ 40% grade), (D) Easy slope (< 25% grade). Peak 3D θ_DE_: peak downhill evacuation 3D excursion angles, CC: cervical collar, MILS: manual in-line stabilization

Figure 5 shows the continuous data gathered for each downhill evacuations (DEs) performed. Despite testing on various terrains including an easy slope (< 25% grade), an intermediate slope (25–40% grade), a steep regular slope (≥ 40% grade) and a steep mogul slope (≥ 40% grade), the peak downhill evacuation 3D excursion angle (Peak 3D θ_DE_) never reached a value above 5°, with little variation over time both with and without a cervical collar. Cervical spine motion recorded during DEs across all experimental conditions were thus considered clinically negligible no matter the use or non-use of CCs.

### Manipulations’ cervical spinal motion (CSM) risk

**Fig. 6:**
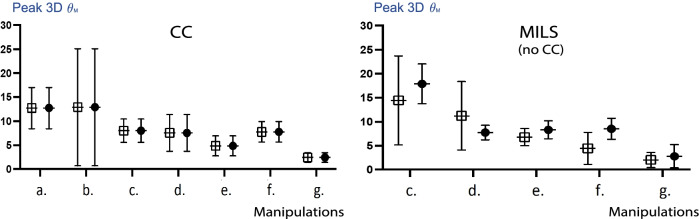
Means and 95% confidence intervals of peak manipulation 3D excursion angles depending on extrication manipulations and cervical collar use. Left: Peak manipulation 3D excursion angles’ means recorded when using a cervical collar (all terrains included); Right: Peak manipulation 3D excursion angles’ means recorded when using manual in-line stabilization (all terrains included). (**a**) Winter clothes removal, (**b**) cervical collar installation, (**c**) logroll to lateral decubitus, (**d**) logroll back to supine, (**e**) move to vacuum mattress midline, (**f**) immobilization on the vacuum mattress, (**g**) transfer to toboggan. •: flat area, □: steep slope, Peak 3D θ_M:_ peak manipulation 3D excursion angle

To identify which manipulation induces the highest CSM risk, extrications were divided into the following events: (a) equipment removal, (b) CC installation, (c) logroll from supine to lateral decubitus, (d) logroll from lateral decubitus back to supine on the mattress, (e) translation on the mattress, (f) immobilization on the mattress, and g) transfer on the toboggan. Figure 6 demonstrates the means of peak manipulation 3D excursion angles (Peak 3D θ_M_). This data suggests a trend towards an increased motion risk during CC installation when extrications are done using a CC. For trials without CC the logroll to lateral decubitus appears to generate the most motion.

## Discussion

This controlled biomechanical study performed in real-life conditions with experienced patrollers suggests the following key points regarding the use of a cervical collar for alpine skiing rescues: (1) Cervical spine motion during extrications with CC compared to MILS without CC are non-inferior according to a 10 degrees non-inferiority hypothesis testing (*p* < 0.05); (2) Time to extrication completion is increased on both terrain conditions tested (*p* < 0.05); (3) Downhill evacuations using a vacuum mattress providing optimal head stabilization produce only very small absolute cervical motion no matter if a CC is used or not (< 5 degrees); (4) When using MILS without CC, special care should be taken at the initiation of the logroll as it showed a higher risk of causing CSM.

Currently, there is no known established amount of movement of an injured cervical spine that would lead to neurological injuries as minimally clinically important difference (MCID) on spinal motion restriction is a challenging and poorly studied topic. The 10 degrees non -inferiority threshold selected to compare the effect of using a CC or MILS in our data analysis can be construed as somewhat arbitrary yet it is aligned with the current paradigm shift of spinal motion restriction (SMR) as opposed to full spinal immobilization [6] and takes into consideration the important variations reported in spinal motions during mobilization and transfer of real or simulated patients. For instance, a recent trial published by McDonald et al. on real victims with a suspected traumatic spinal injury equipped with inertial measurements units (IMUs) underlined the wide range of multi-plane head-neck motion (from 7.2° to 82.1°) [47]. Their results suggest that our < 10 degrees threshold would be conservative. Remarkably, authors of this study also stated that patient compliance (anxiety, intoxication, pain, etc.) was significantly more related to cervical spine motions recorded than the motion restriction protocol used. Another trial using 24 healthy volunteers to compare cervical flexion/extension, rotation, and lateral flexion with either an improvised fleece collar or a standard cervical collar also used a 10 degree non inferiority threshold at limiting the designated motions [48]. Furthermore, a 1975 landmark study previously suggested 11 degrees at a single cervical vertebrae level as a clinical threshold for instability [49]. It must be noted that this value was obtained on cadaveric ligamentous simulated injuries and that it remains only an extrapolation from biomechanical data, it was however long considered a significant threshold and used clinically to rule out cervical instability on flexion–extension radiographs of trauma patients [50]. Considering this threshold relates to a single vertebrae level in a single motion axis whereas the data collected in the presented study refers to all cervical levels combined (seven) in 3 dimensional motion axis it may suggest that the overall CSM 10 degrees non-inferiority testing used is reasonable. Noteworthy, this 11 degrees value is fading in recent literature as MRI and CT scans have similar diagnostic results to traditional flexion–extension radiographs without the risks of mobilizing a potentially unstable cervical spine [51].

This study reinforces emerging guidelines previously discussed supporting the removal of systematic cervical collars use from first-aid protocols to save critical time [7, 9–11, 30, 52, 53]. A recent study on maximum movement and cumulative movement of the head measured with IMUs during MVA self-extrications simulation showed that total travel motion is similar across self-extricating healthy volunteers with and without a collar [54].

According to our results and considering the previous discussion on the minimally clinically important difference (MCID) of spinal motion restriction, the motion reduction expected with a cervical collar, in the setting tested, is suspected to be not clinically significant. One must also consider that presumed benefit from CC use would have to outweigh its drawbacks: required installation time (approximating a 60% longer total extrication time), victim's cold exposure, possible airway complications, pressure ulcers, etc. Interestingly, some outlier results shown in Fig. 3, both during MILS and CC manipulations, as well as the subjective feedback from the patrollers support that minimal distractions may induce important cervical motions. Concentration during the whole EXs is a key factor to reduce CSM regardless of the use or not of a CC. Regarding the downhill evacuations it is interesting to note that once a vacuum mattress properly stabilizes the head the cervical collar appears unnecessary.

A caveat to this is that our results, while obtained in real ski rescue conditions on two different surfaces including a 45-degree slope, can’t be generalized to all rescue conditions and environments. There might be a protective effect of using CC for ski rescues performed under more complex and hostile environments where MILS could either be hardly properly executed or not maintained long enough. Lack of appropriate resources in terms of manpower or level of experience are other factors to consider. As such, cervical collar application should be individually assessed depending on each rescue situation, according to the terrain, weather, urgency, health care providers available, etc.

Our study stands out owing to its use of a high-fidelity mannequin and real-life situation data collection. Few biomechanical studies on spinal stabilization are designed in real-life settings and these are essentially focused on ambulance transport or vehicle extrication [55, 56]. There is no study on spinal stabilization completed in real mountain rescue conditions that we are aware of. A thorough sample of slope grades for both EXs and DEs were also carefully chosen to realistically portray the average alpine skiing rescues happening in Northeastern America. The high-fidelity mannequin used in this study is also an interesting tool to further study cervical spine motion restriction devices and protocols considering SMR studies are known to be challenging for convenient accurate data collection [57]. Current literature relies on data obtained with conscious volunteers or with cadavers using various external motion capture systems. Healthy volunteer studies mostly evaluate the efficacity of collars according to their reduction of active movements [8, 58]. Cadaveric studies enable the possibility to surgically induce a cervical instability prior to spinal stabilization tests however, they are not suitable for real-life situations data collection. As for external motion capture systems they have practicality issues for real life situation data collection that are not present when using an integrated system. These reasons combined led to the development and use of this mannequin for such experimental studies. For future perspectives, this mannequin could also be used for prehospital health care providers training through high-fidelity simulations.

Numerous limitations of this study must be outlined. First, this was a simulation study performed in real-life ski rescue conditions with an instrumented mannequin simulating an unconscious human and measuring cervical motions. The observations made are for a small sample of trials under specific rescue conditions in a small sample of experienced ski patrollers. Any conclusive answers as to the clinical impact of using or not a cervical collar for ski rescue will require evidence from controlled studies and or observational studies. Second, ski patrollers were all volunteers and experts with more than 9 years of experience. One might fairly argue that some less experienced patrollers could induce more movements when stabilizing the cervical spine without the assistance of a cervical collar. However, the same could be true for hazardous motion during the cervical collar installation which would void its benefits. Third, as a limitation it should be noted that our data collection could not be blinded, and patrollers were vulnerable to the Hawthorne effect as they were indeed aware of the use or not of the collar and knew they were observed. Fourth, our number of repetitions and Peak 3D θ_EX_ values remain small (EXs = 32). Although, as discussed previously, the 10 degrees margin reported is proposed to be safe, perhaps a higher number of repetitions could demonstrate non-inferiority within a 5 degrees margin thus strengthening evidence. Finally, the mannequin used in this study has some limitation in terms of external validity with respect to a real victim as no validation publication exists comparing its cervical motion compared to a human’s. A validation could provide interesting information but would be technically challenging considering the variety of human cervical physiognomy in terms of length, weight, potential vertebral ankylosis on aging spine, etc. Noteworthy, the mannequin overall cervical motion realism was confirmed by multiple first aid and medical providers and its efficiency in sub-zero temperatures was also tested before this trial.

## Conclusion

The biomechanical benefits of cervical collars in terms of spinal motion restriction (SMR) during a simulated alpine rescue in comparison to MILS seem to be marginal. Considering the difficulties of applying cervical collars under these rescue conditions, systematic use of the cervical collar by first aid providers during alpine skiing rescues should be questioned. With experienced ski patrollers, the application of MILS during alpine ski rescues may achieve similar levels of SMR to what is observed with a cervical collar.

## Supplementary Information

**Additional file 1:** Demonstration video of simulation mannequin.

**Additional file 2:** Technical brief on design of simulation mannequin.

## Data Availability

The data presented in this study are available on request from the corresponding author.
