# Supplementary material for: Removal of the cervical collar from alpine rescue protocols? A biomechanical non-inferiority trial in real-life mountain conditions
Source: Scand J Trauma Resusc Emerg Med. 2022 Jun 27;30:42. doi: 10.1186/s13049-022-01031-3 (PMC9235139; doi:10.1186/s13049-022-01031-3)
Supplement: Supplementary file 2 — Additional file 2: Technical brief on design of simulation mannequin. [file 13049_2022_1031_MOESM2_ESM.pdf]

US 20210192977A1

(19) United States
(12) Patent Application Publication
BOISSY et al.
(10) Pub. No.: US 2021/0192977 A1
(43) Pub. Date: Jun. 24, 2021

(54) **SYSTEM FOR SIMULATING CERVICAL SPINE MOTIONS**

(71) Applicant: **SOCIETE DE COMMERCIALISATION DES PRODUITS DE LA RECHERCHE APPLIQUEE SOCPRA SCIENCES ET GENIE S.E.C.**, Sherbrooke (CA)

(72) Inventors: **Patrick BOISSY**, Sherbrooke (CA); **Mathieu HAMEL**, Magog (CA); **Francois CABANA**, Saint-Denis-de-Brompton (CA); **Karina LEBEL**, Sherbrooke (CA)

(21) Appl. No.: **16/757,437**

(22) PCT Filed: **Oct. 22, 2018**

(86) PCT No.: **PCT/CA2018/051334**

§ 371 (c)(1),
(2) Date: **Apr. 20, 2020**

Related U.S. Application Data

(60) Provisional application No. 62/575,016, filed on Oct. 20, 2017.

Publication Classification

(51) **Int. Cl.**
G09B 23/32 (2006.01)
G09B 23/30 (2006.01)

(52) **U.S. Cl.**
CPC ***G09B 23/32*** (2013.01); ***G09B 23/303*** (2013.01)

(57) **ABSTRACT**

A neck mechanism for a mannequin comprises three or more joint units serially connected to provide joints for three or more rotational degrees of freedom (DOF). A rotational axis of a first DOF is configured to be aligned with a lateral axis of the mannequin. A rotational axis of a second DOF is configured to be aligned with an anterior-posterior axis of the mannequin. A rotational axis of a third DOF is configured to be aligned with a cranial-caudal axis of the mannequin. A bottom one of the joint units is adapted to be connected to a torso of the mannequin, and a top one of the at least three joint units is adapted to be connected to a skull. The mannequin may also have a skull connected to the top one of the joint units, and a trunk connected to the bottom one of the joint units. A system for simulating cervical spine motions is also provided.

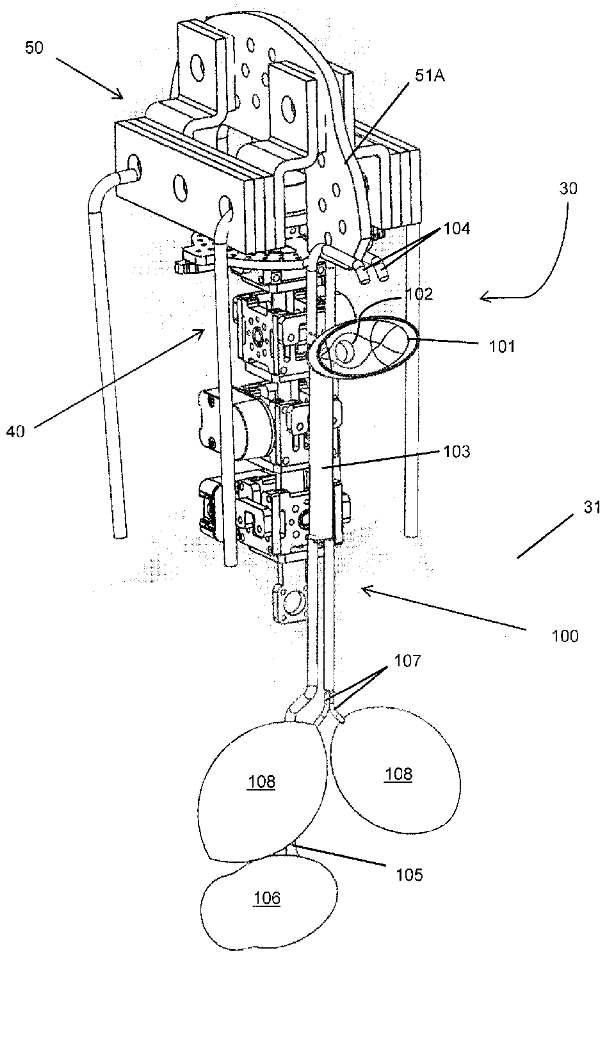

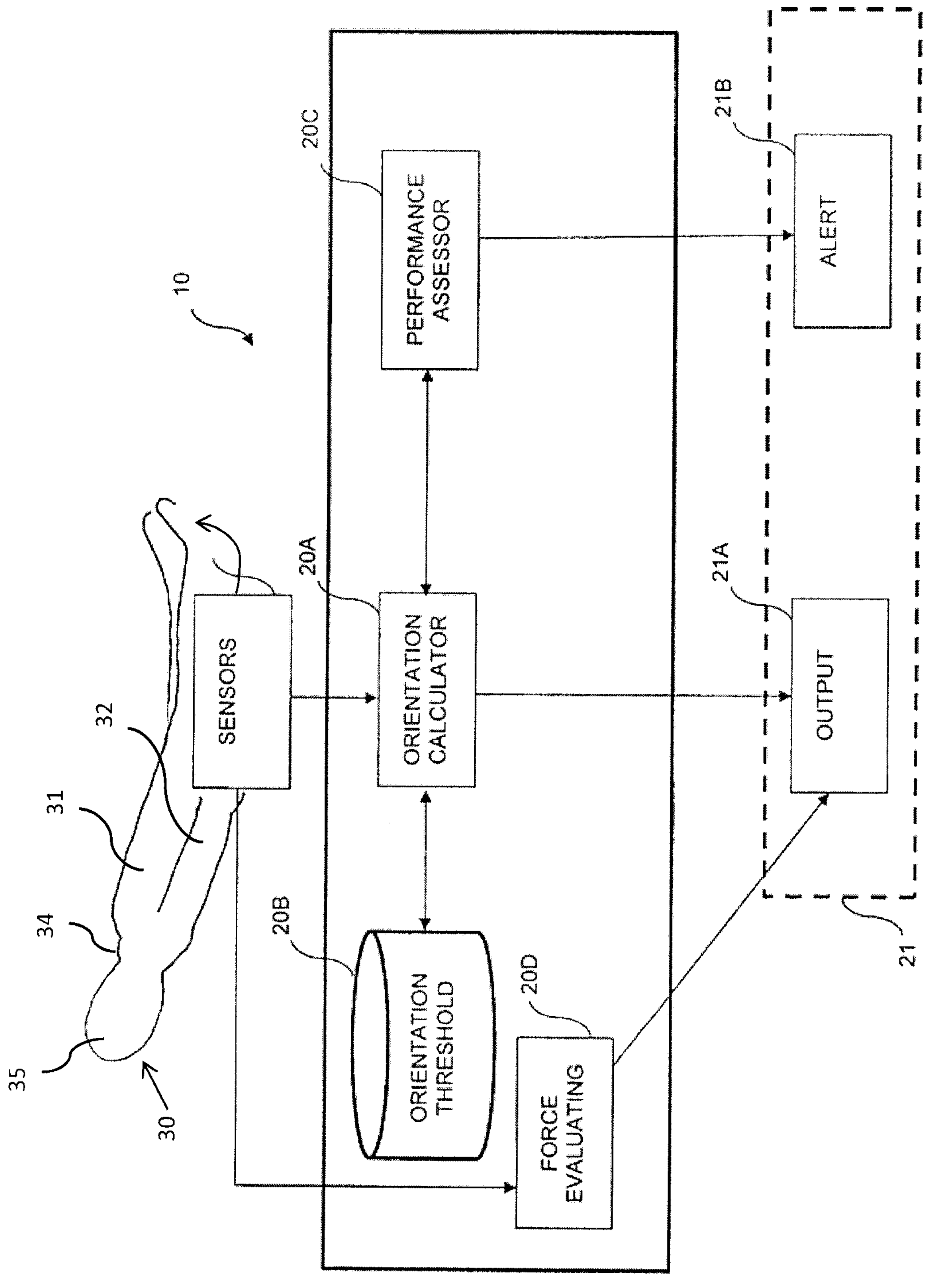
10
20C
PERFORMANCE ASSESSOR
21B
ALERT
20A
ORIENTATION CALCULATOR
SENSORS
21A
OUTPUT
32
31
34
35
30
20B
ORIENTATION THRESHOLD
20D
FORCE EVALUATING
21

FIG. 1

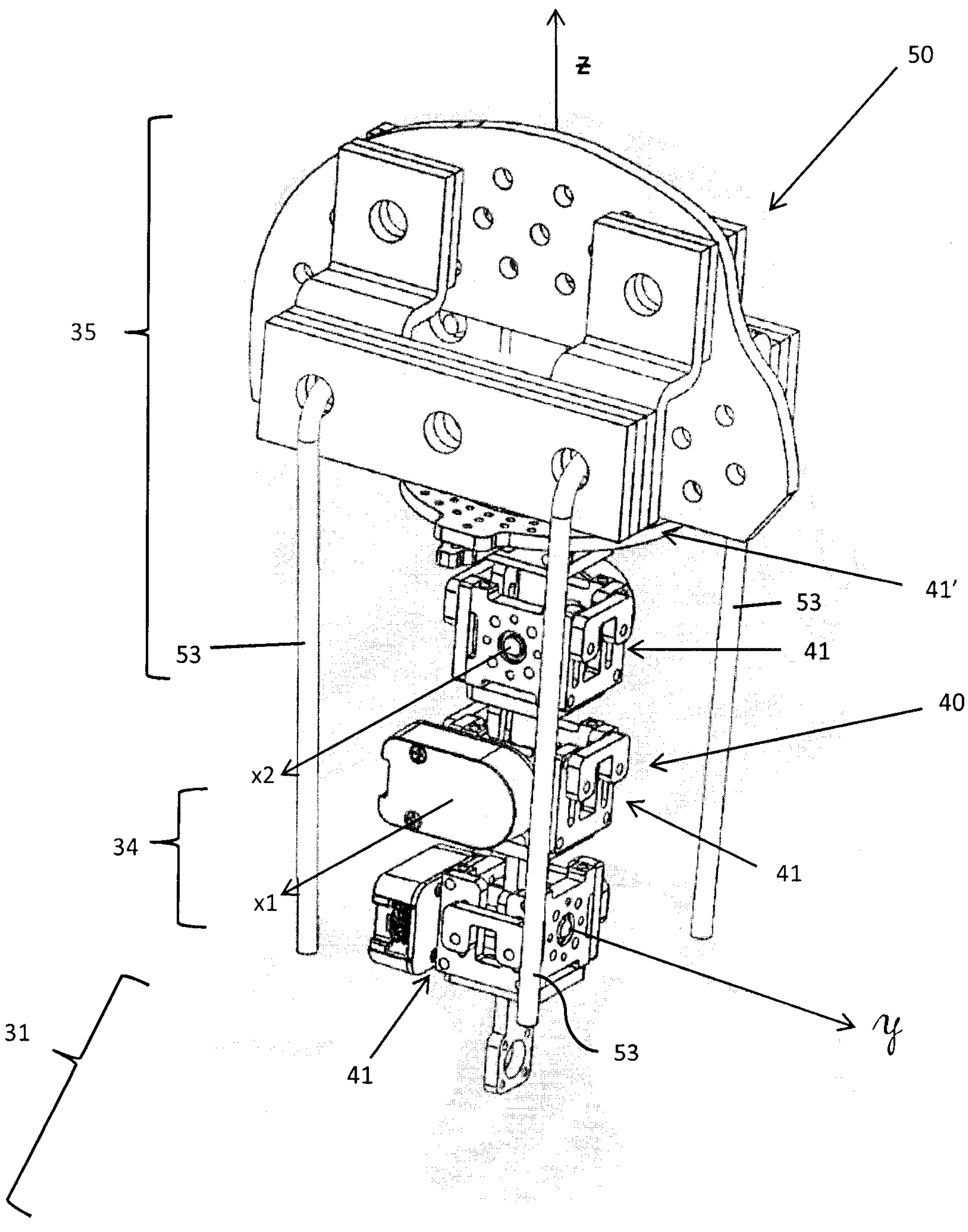

FIG. 2

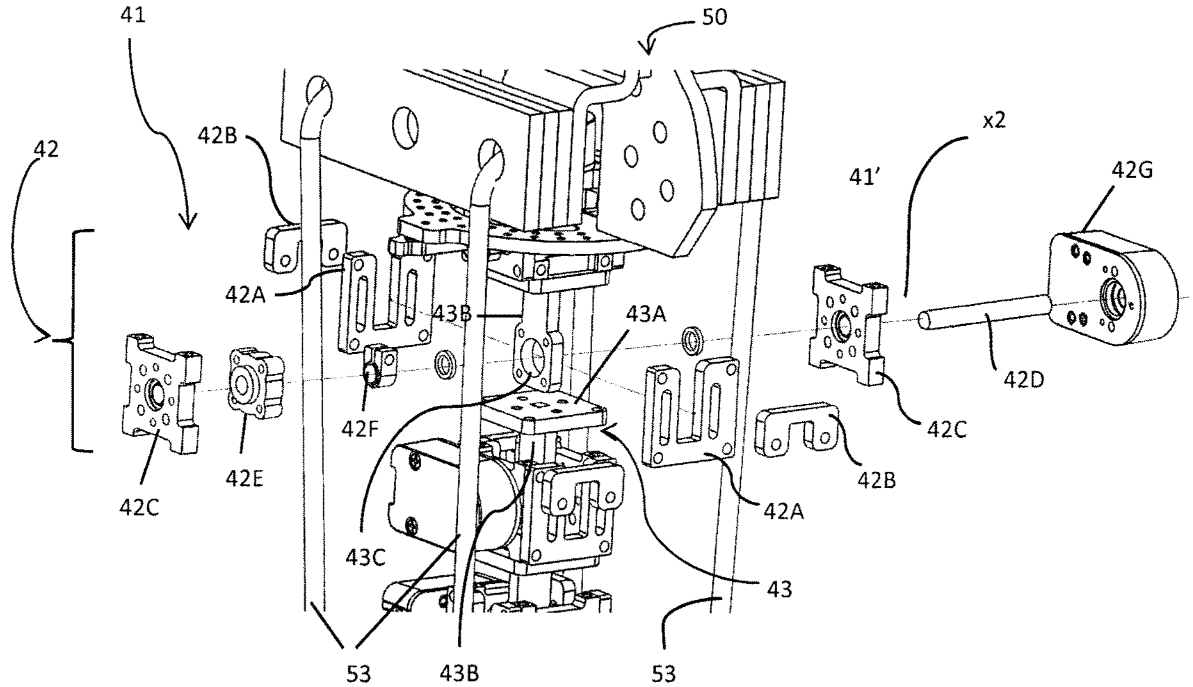
41
50
42B
42
x2
41'
42G
42A
43B
43A
42F
42C
42D
42C
42E
42B
42A
43C
43
53
43B
53

FIG. 3

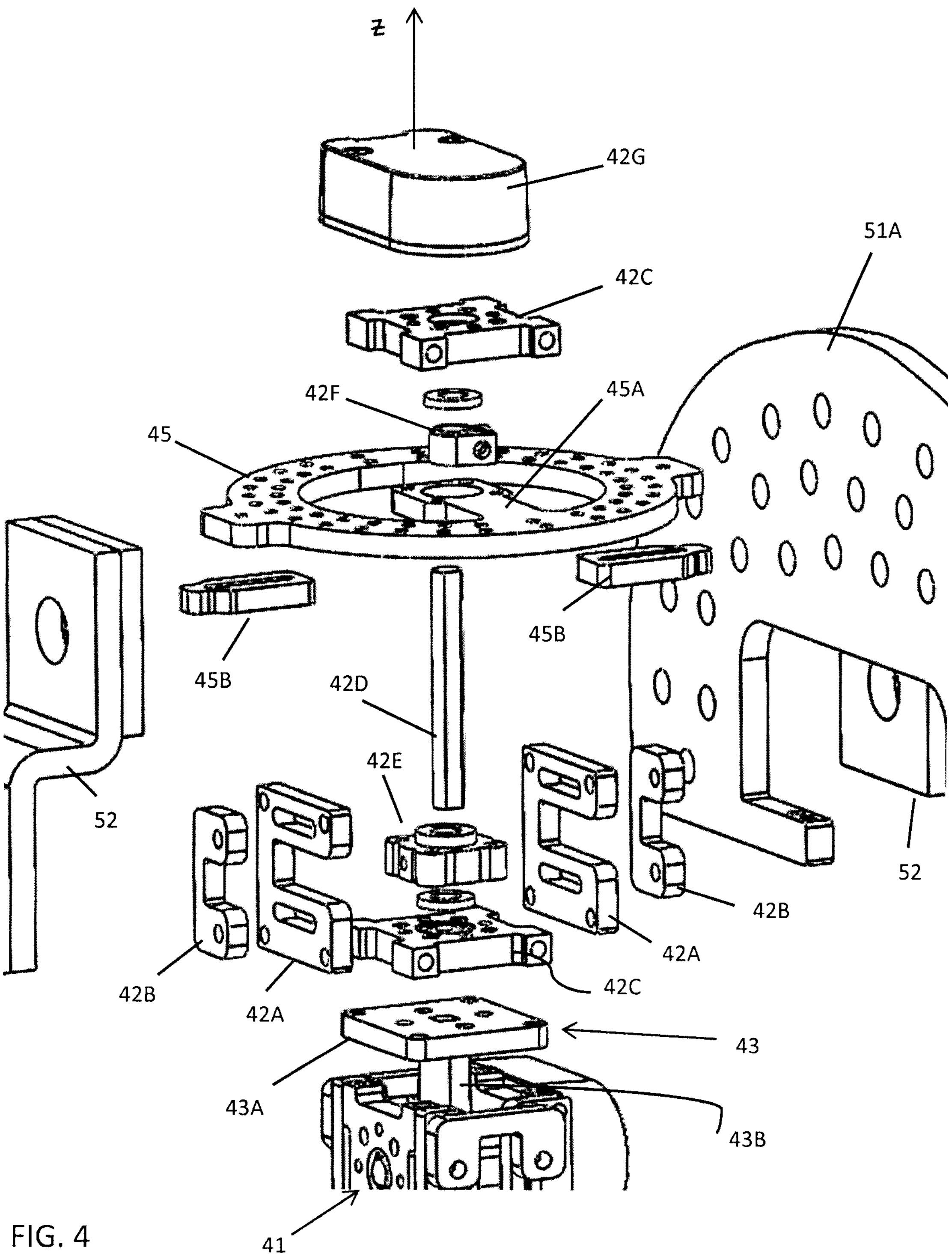
Z
42G
51A
42C
42F
45A
45
45B
45B
42D
42E
52
52
42B
42A
42C
42B
42A
43
43A
43B
41

FIG. 4

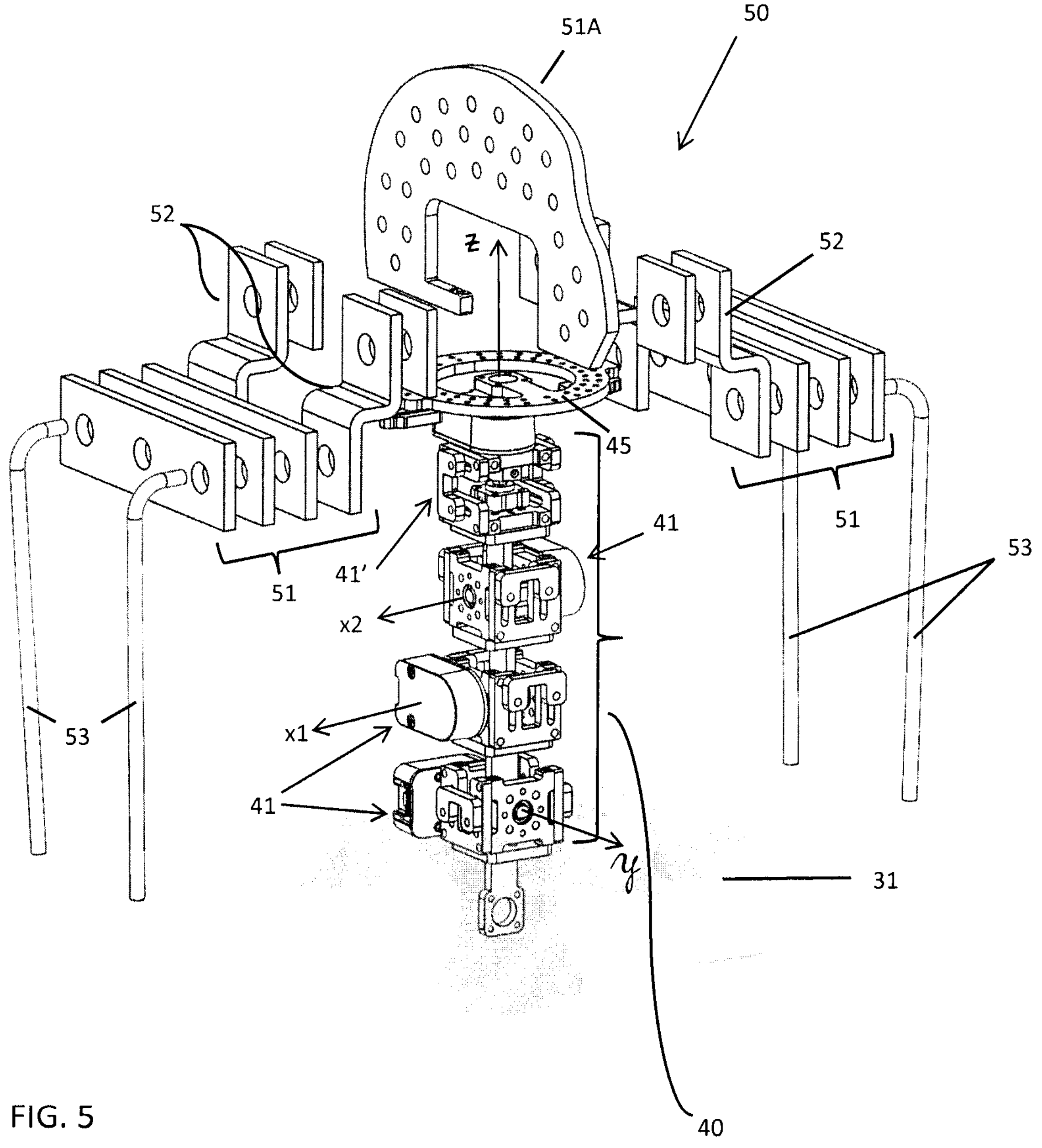

FIG. 5

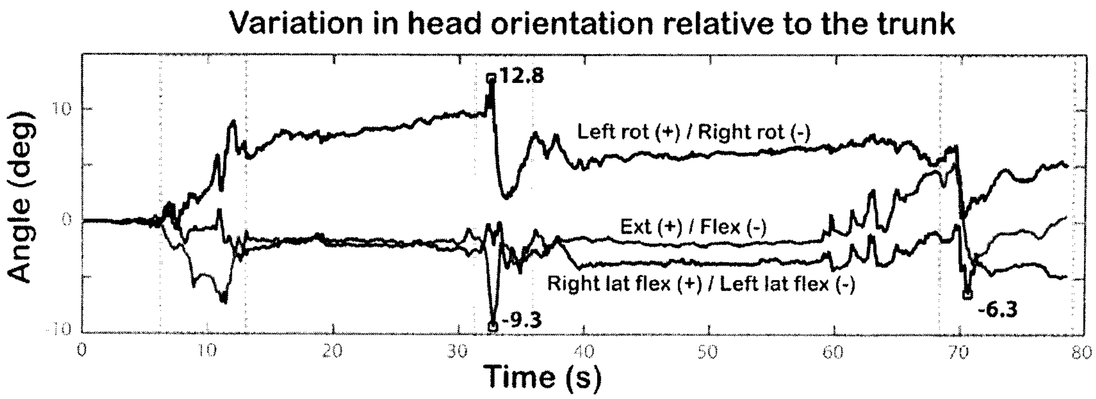
Variation in head orientation relative to the trunk
12.8
Left rot (+) / Right rot (-)
Ext (+) / Flex (-)
Right lat flex (+) / Left lat flex (-)
-9.3
-6.3
Angle (deg)
10
0
-10
0
10
20
30
40
50
60
70
80
Time (s)

FIG. 6

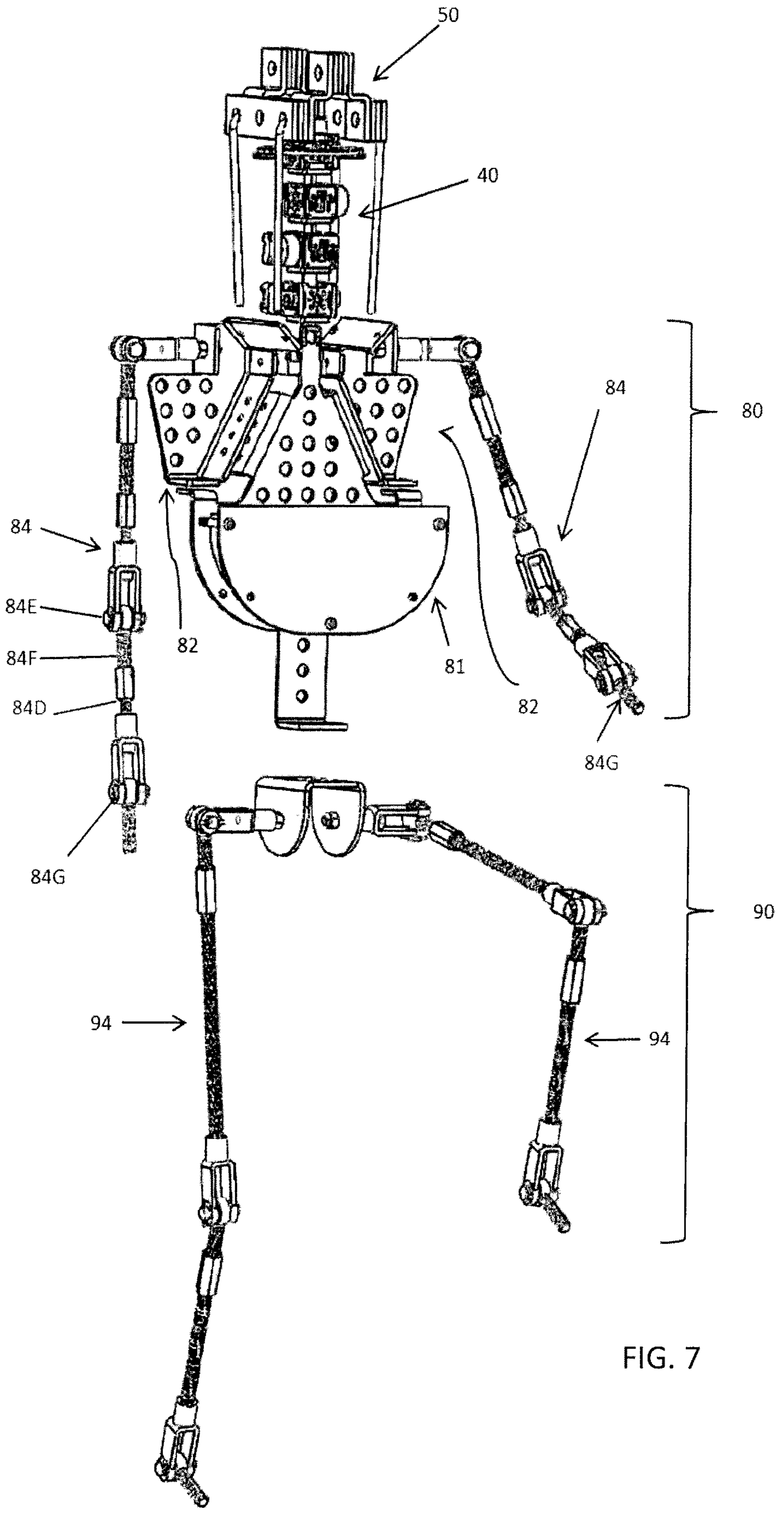

FIG. 7

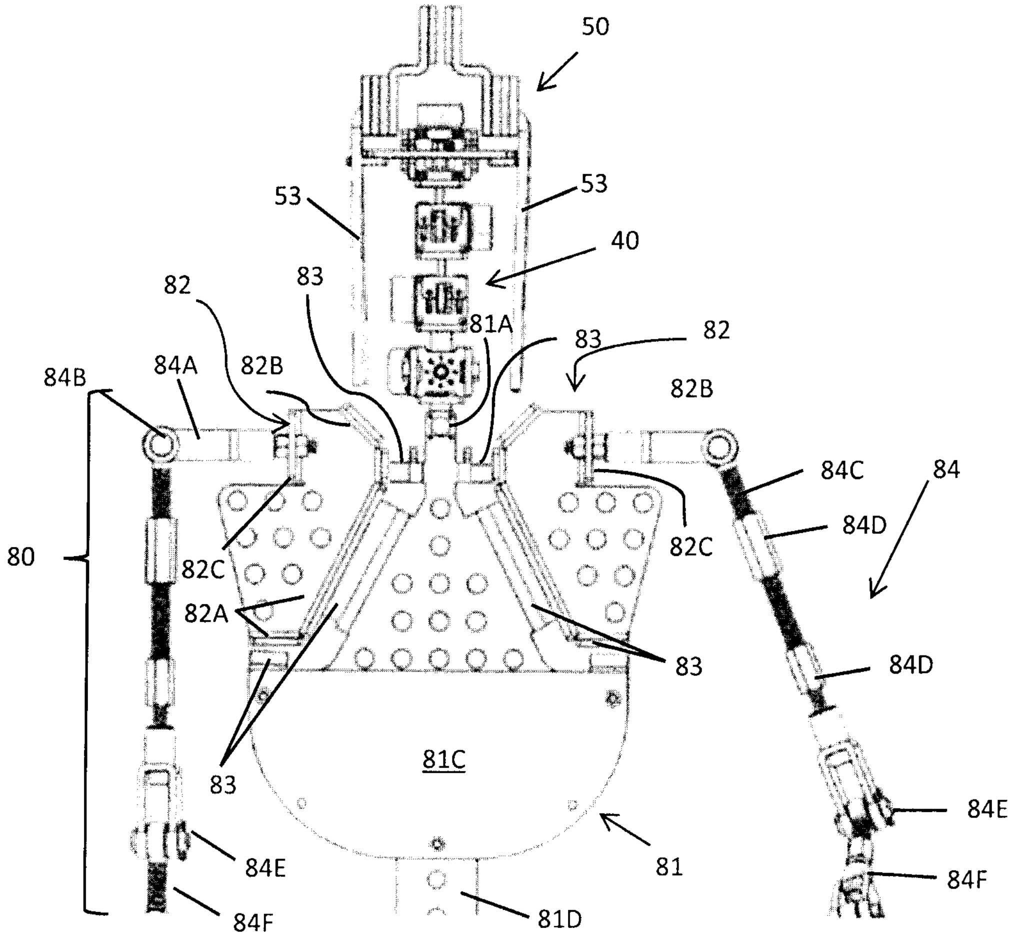
50
53
53
40
82
83
81A
83
82
84B
84A
82B
82B
84C
84
84D
80
82C
82C
82A
83
84D
83
81C
84E
81
84E
84F
84F
81D

FIG. 8

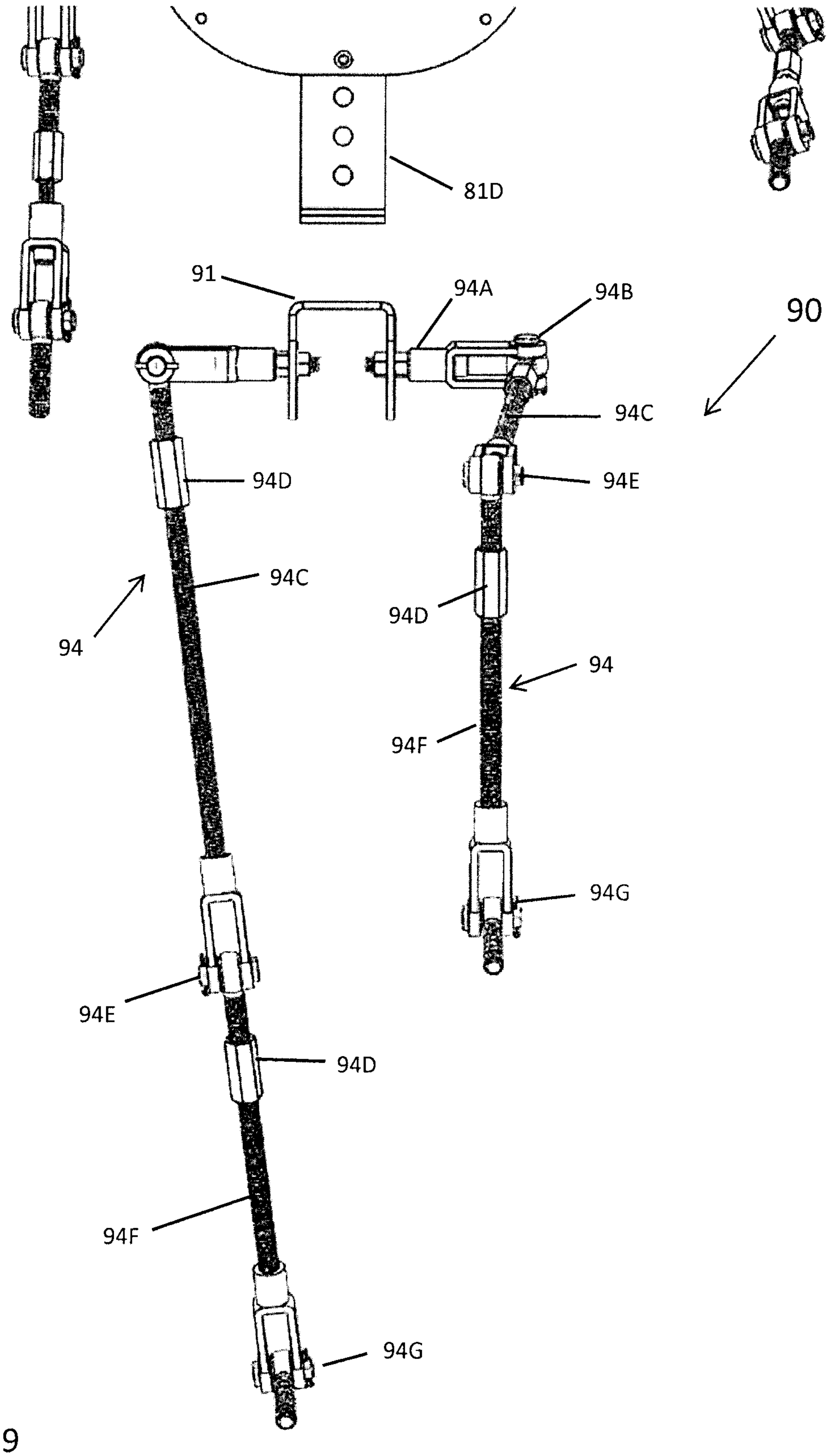
81D
91
94A
94B
90
94C
94D
94E
94C
94
94D
94
94F
94G
94E
94D
94F
94G

FIG. 9

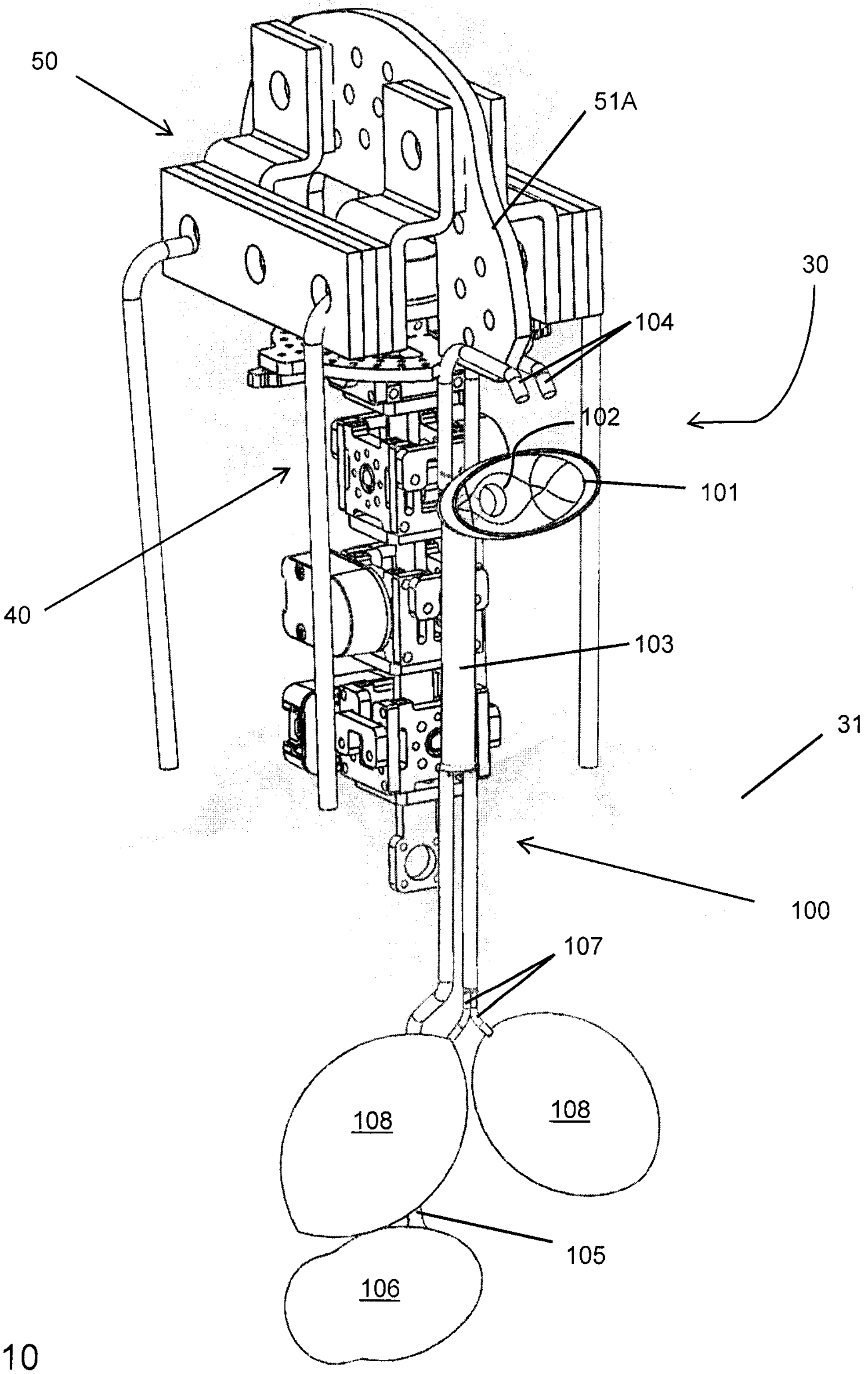

FIG. 10

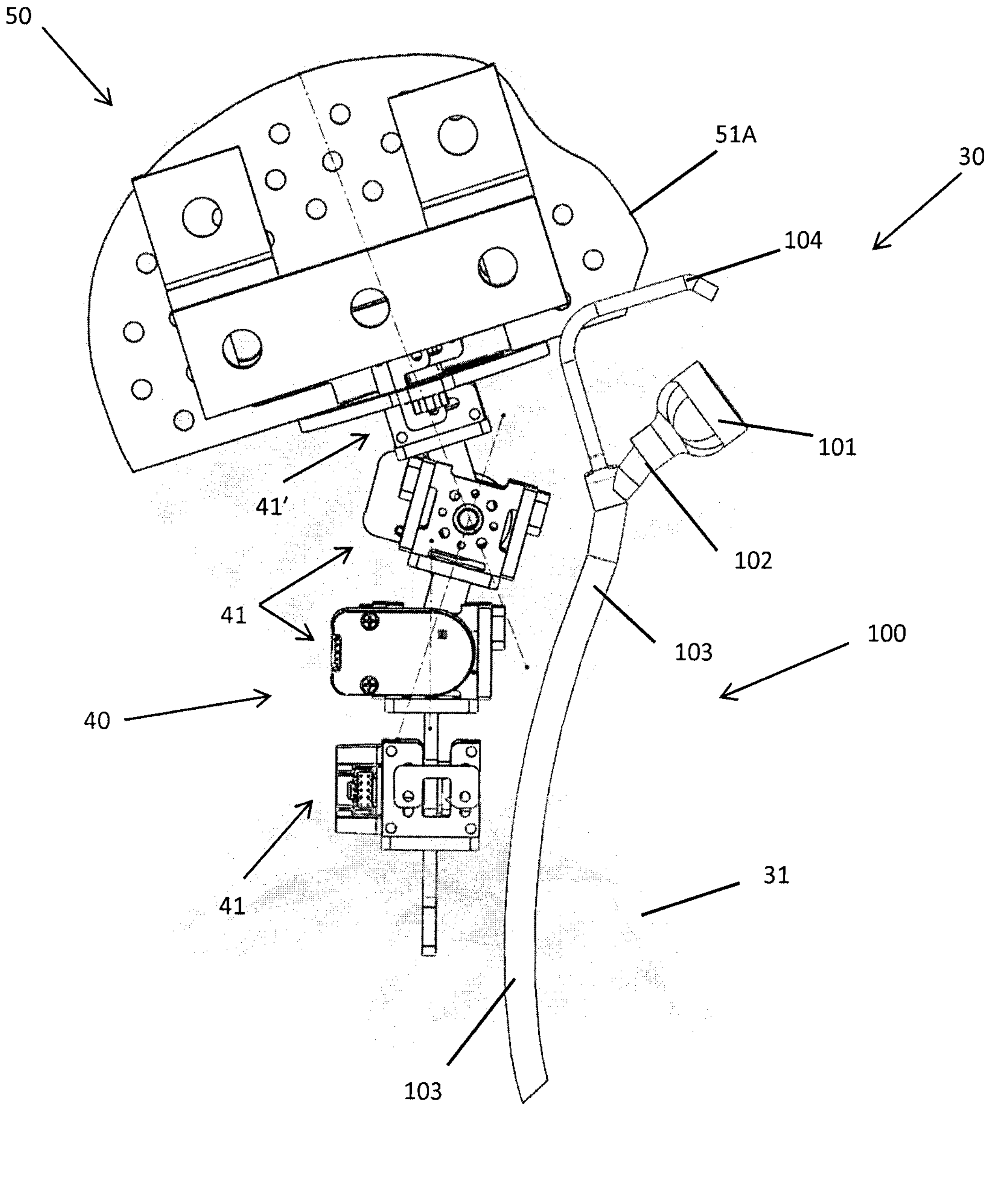

FIG. 11

SYSTEM FOR SIMULATING CERVICAL SPINE MOTIONS

CROSS-REFERENCE TO RELATED APPLICATION

[0001] The present application claims the priority of U.S. Patent Application No. 62/575,016, filed on Oct. 20, 2017 and incorporated herein by reference.

TECHNICAL FIELD

[0002] The present disclosure pertains to a method and system for evaluating cervical spine stabilization in patient transfer training, and for evaluating tracheal intubation and/or direct laryngoscopy maneuvering techniques.

BACKGROUND OF THE ART

[0003] Patients with suspected spinal cord injuries undergo numerous transfers throughout treatment and care. Effective cervical spine (c-spine) stabilization is crucial to minimize the impacts of the suspected injury. Healthcare professionals are trained to perform transfers using simulation. However, the feedback on the maneuvers is often subjective, i.e., it is performed by an on-site evaluator that visually assesses whether the maneuvers are adequate.

[0004] Also, c-spine motion may result by the difficulty of rescuers to maintain head and trunk alignment during a rotation step of a transfer motion, in addition to the difficulty in initiating specific phases of the motion synchronously when multiple rescuers perform transfers. It may be difficult for an evaluator to do a proper visual assessment considering rapidity of a rotation step and number of participants.

SUMMARY

[0005] It is an aim of the present disclosure to provide a novel system for simulating cervical spine motions.

[0006] It is a further aim of the present disclosure to use the system for simulating cervical spine motions for evaluating tracheal intubation maneuvers and/or direct laryngoscopy maneuvers.

[0007] Therefore, in accordance with a first embodiment of the present disclosure, there is provided a neck mechanism for a mannequin comprising: at least three joint units serially connected to provide joints for at least three rotational degrees of freedom (DOF), with a rotational axis of a first DOF configured to be aligned with a lateral axis of the mannequin, a rotational axis of a second DOF configured to be aligned with an anterior-posterior axis of the mannequin, and a rotational axis of a third DOF configured to be aligned with a cranial-caudal axis of the mannequin, wherein a bottom one of the at least three joint units is adapted to be connected to a torso of the mannequin, and a top one of the at least three joint units is adapted to be connected to a skull.

[0008] Further in accordance with the first embodiment, for example, the bottom one of the at least three joint units is adapted to be connected to the torso of the mannequin with the second DOF.

[0009] Still further in accordance with the first embodiment, for example, the top one of the at least three joint units is adapted to be connected to a skull by the third DOF.

[0010] Still further in accordance with the first embodiment, for example, four joint units, provide concurrently four rotational DOFs, wherein a rotational axis of a fourth DOF is configured to be aligned with the lateral axis of the mannequin.

[0011] Still further in accordance with the first embodiment, for example, the rotational axes of the first DOF and of the fourth DOF are parallel and spaced apart to another.

[0012] Still further in accordance with the first embodiment, for example, the rotational axes of the first DOF and of the fourth DOF are parallel and spaced apart relative to the rotational axis of the second DOF.

[0013] Still further in accordance with the first embodiment, for example, a rotary motion sensor is at each said joint unit.

[0014] In accordance with a second embodiment of the present disclosure, there is provided a mannequin comprising: the neck mechanism as described above; a skull connected to the top one of the at least three joint units; and a trunk connected to the bottom one of the at least three joint units.

[0015] Further in accordance with the second embodiment, for example, the trunk has a plurality of metal plates emulating a volume and/or a weight of an anatomical skull.

[0016] Still further in accordance with the second embodiment, for example, the skull has a disk ring connected to a shaft of the top one of the at least three joint units.

[0017] Still further in accordance with the second embodiment, for example, elastics connect the skull to the trunk.

[0018] Still further in accordance with the second embodiment, for example, an airway simulator apparatus has at least one tube defining at least one opening at a level of a face of the mannequin, and being in fluid communication with at least one expandable balloon in the trunk.

[0019] Still further in accordance with the second embodiment, for example, the airway simulator apparatus includes a mouthpiece connected to the at least one tube at the at least one opening.

[0020] Still further in accordance with the second embodiment, for example, the at least one tube includes at least one nose tube having an opening defining a nostril.

[0021] Still further in accordance with the second embodiment, for example, the at least one tube diverges into at least two tracheal tubes, with one said expandable balloon at an end of each said tracheal tube.

[0022] Still further in accordance with the second embodiment, for example, the at least one tube diverges into an oesophageal tube, with one said expandable balloon at an end of the oesophageal tube.

[0023] Still further in accordance with the second embodiment, for example, the at least one tube is flexible tubing.

[0024] Still further in accordance with the second embodiment, for example, the at least one tube is connected to the skull.

[0025] Still further in accordance with the second embodiment, for example, the trunk comprises an articulated skeleton.

[0026] Still further in accordance with the second embodiment, for example, at least the neck mechanism, the skull and the trunk are covered by a skin membrane.

[0027] In accordance with a third embodiment of the present disclosure, there is provided a system for simulating cervical spine motions, the system comprising: a mannequin having a neck mechanism between a torso and a skull, the neck mechanism having at least three degrees of freedom (DOF); sensors to detect cervical spine orientation changes;

a processing unit having an orientation calculator module to quantify the cervical spine orientation changes from readings of the sensors, and a performance assessor module to assess the cervical spine motions using the quantified cervical spine orientation changes; and an output for outputting an assessment and/or the cervical spine orientation changes.

[0028] Further in accordance with the third embodiment, for example, the neck mechanism has at least three joint units serially connected to provide joints for at least three rotational degrees of freedom (DOF), with a rotational axis of a first DOF configured to be aligned with a lateral axis of the mannequin, a rotational axis of a second DOF configured to be aligned with an anterior-posterior axis of the mannequin, and a rotational axis of a third DOF configured to be aligned with a cranial-caudal axis of the mannequin, wherein a bottom one of the at least three joint units is adapted to be connected to a torso of the mannequin, and a top one of the at least three joint units is adapted to be connected to a skull.

[0029] Still further in accordance with the third embodiment, for example, the bottom one of the at least three joint units is adapted to be connected to the torso of the mannequin with the second DOF.

[0030] Still further in accordance with the third embodiment, for example, the top one of the at least three joint units is adapted to be connected to a skull by the third DOF.

[0031] Still further in accordance with the third embodiment, for example, four joint units, provide concurrently four rotational DOFs, wherein a rotational axis of a fourth DOF is configured to be aligned with the lateral axis of the mannequin.

[0032] Still further in accordance with the third embodiment, for example, the rotational axes of the first DOF and of the fourth DOF are parallel and spaced apart to another.

[0033] Still further in accordance with the third embodiment, for example, the rotational axes of the first DOF and of the fourth DOF are parallel and spaced apart relative to the rotational axis of the second DOF.

[0034] Still further in accordance with the third embodiment, for example, the sensors include a rotary motion sensor at each said joint unit.

[0035] Still further in accordance with the third embodiment, for example, the skull is connected to the top one of the at least three joint units; and the trunk is connected to the bottom one of the at least three joint units.

[0036] Still further in accordance with the third embodiment, for example, the trunk has a plurality of metal plates emulating a volume and/or a weight of an anatomical skull.

[0037] Still further in accordance with the third embodiment, for example, the skull has a disk ring connected to a shaft of the top one of the at least three joint units.

[0038] Still further in accordance with the third embodiment, for example, elastics connect the skull to the trunk.

[0039] Still further in accordance with the third embodiment, for example, an airway simulator apparatus has at least one tube defining at least one opening at a level of a face of the mannequin, and being in fluid communication with at least one expandable balloon in the trunk, the performance assessor module assessing an airway alignment for direct laryngoscopy and/or tracheal intubation using the quantified cervical spine orientation changes.

[0040] Still further in accordance with the third embodiment, for example, the airway simulator apparatus includes a mouthpiece connected to the at least one tube at the at least one opening.

[0041] Still further in accordance with the third embodiment, for example, the at least one tube includes at least one nose tube having an opening defining a nostril.

[0042] Still further in accordance with the third embodiment, for example, the at least one tube diverges into at least two tracheal tubes, with one said expandable balloon at an end of each said tracheal tube.

[0043] Still further in accordance with the third embodiment, for example, the at least one tube diverges into an oesophageal tube, with one said expandable balloon at an end of the oesophageal tube.

[0044] Still further in accordance with the third embodiment, for example, the at least one tube is flexible tubing.

[0045] Still further in accordance with the third embodiment, for example, the at least one tube is connected to the skull.

[0046] Still further in accordance with the third embodiment, for example, the trunk comprises an articulated skeleton.

[0047] Still further in accordance with the third embodiment, for example, at least the neck mechanism, the skull and/or the trunk are covered by a skin membrane

DESCRIPTION OF THE DRAWINGS

[0048] FIG. **1** is a block diagram of a system for simulating cervical spine motions;

[0049] FIG. **2** is a perspective view of a neck mechanism and skull of a mannequin of the system of the present disclosure;

[0050] FIG. **3** is an exploded view of a joint unit of the neck mechanism of FIG. **2**;

[0051] FIG. **4** is an exploded view of another of the joint units of the neck mechanism of FIG. **2**;

[0052] FIG. **5** is an exploded view of a skull of the neck mechanism of FIG. **2**;

[0053] FIG. **6** is a graph showing an output of the system of FIG. **1**; and

[0054] FIG. **7** is a schematic view of a mannequin skeleton that may be part of the system of FIG. **1**;

[0055] FIG. **8** is an enlarged schematic view of a torso of the mannequin skeleton of FIG. **7**;

[0056] FIG. **9** is an enlarged schematic view of a lower body of the mannequin skeleton of FIG. **7**;

[0057] FIG. **10** is a perspective view of the neck mechanism and skull of the mannequin of FIG. **2**, with an airway simulator apparatus; and

[0058] FIG. **11** is a side view of the assembly of the airway simulator apparatus, the neck mechanism and skull of the mannequin.

DETAILED DESCRIPTION

[0059] Referring to the drawings and more particularly to FIG. **1**, there is illustrated a system for simulating cervical spine motions at **10**, as resulting from cervical spine manipulations for example. The system **10** is of the type having a processing unit **20** used to quantify the manipulations of a mannequin **30**, also known as a dummy, etc. The processing unit **20** is of the type having a non-transitory computer-readable memory communicatively coupled to it and comprising computer-readable program instructions executable by the processing unit **20** to perform numerous functions related to the simulation of cervical spine motions. As shown in FIG. **1**, the processing unit **20** may have various modules

to perform these functions. The system **10** receives data from sensors generically shown as A in FIG. **1** but described in further detail hereinafter. The sensors A may be any appropriate type of sensor to measure various movements of the mannequin **30** and other parameters such as forces applied to mannequin **30**. An interface **21** may be operatively connected to the processing unit **20** to output quantitative data representative of the transfer manipulations, or airway management during tracheal intubation and/or direct laryngoscopy maneuvers, and may communicate with the operators of the system **10** to warn or alarm them of excessive or improper manipulations. The interface **21** may be a monitor, screen, tablet, etc.

[0060] The processing unit **20** may have an orientation calculator module **20**A receiving the data from the sensors A. The orientation calculator module **20**A may determine orientation variations sustained by the neck of the mannequin **30** during manipulations. For example, the orientation calculator module **20**A may quantify variations in flexion angle values about one or more flexion axes, about lateral axes of the mannequin **30**. The orientation calculator module **20**A may also quantify lateral flexions as well, i.e., about an anterior-posterior axis of the mannequin **30**. As yet another orientation value that may be calculated by the orientation calculator module **20**A, the rotation may be obtained as well, namely an orientation of a skull relative to a torso along a vertical or cranial-caudal axis of the mannequin **30**. All of these variations of angle values may be in the form of angular rates of change about various axes.

[0061] The orientation calculator module **20**A may provide an output **21**A as a visual display on the interface **21**, or in the form of a data file for any given training session. In accordance with an embodiment, the output **21**A is in the form of the graph of FIG. **6** to indicate the angle or angular rates of change for a user to get a quantitative assessment of manipulations being performed. The graph of FIG. **6** shows a timescale which can be matched with data pertaining to the various manipulations such that a user may see the angles and angular rates of change resulting from various manipulations.

[0062] The processing unit **20** may be programmed with an orientation threshold database **20**B so as to determine what constitutes permitted versus excessive manipulations. Hence, the orientation calculator module **20**A may provide measured angular rates of change and receive threshold values from the orientation threshold database **20**B. A performance assessor **20**C may then determine whether the movements performed exceed the values programmed into the orientation threshold database **20**B, in which case it may be determined that an excessive or improper transfer manipulation of the mannequin **30** has been performed. It may also or alternatively be determined that an excessive or improper intubation or laryngoscopy manipulation of the mannequin **30** has been performed, for instance by the mannequin **30** being too far aligned related to a sniffing position. The performance assessor module **20**C may therefore indicate in real time that manipulations have been improper.

[0063] The performance assessor module **20**C, when identifying an excessive or improper manipulation by measured values exceeding beyond those of the orientation threshold database **20**B, may alert the operator of the system **10** via the alert **21**B of the interface **21**, or may provide quantitative data relative to accepted values, such as a sniffing position. This is an advantage over methods in which the quantitative data is provided at a later point, in that corrective measures may be taken right away to practice by re-manipulating the mannequin **30** for a proper manipulation. Moreover, the orientation threshold database **20**B may have various thresholds to provide more than a binary "proper" vs "excessive" assessment. For example, preliminary signals may be emitted to warn the operators of an impending excessive manipulation, for the operators to correct their movements, for instance by slowing down manipulations, and/or by reorienting the mannequin **30**. Such system interventions may provide real time feedback to the operators during training, for the operators to be capable of understanding the manipulations that are not done correctly.

[0064] The processor unit **20** may also have a force evaluating module **20**D receiving signals from the sensors A to calculate the forces to which the mannequin **30** is exposed. For example, the sensors A may include inertial sensors (e.g., accelerometers) producing data indicative of the forces sustained by the mannequin **30** during the manipulations. The sensors A may include pressure sensors (e.g., manometers) for an airway simulator apparatus, as detailed hereinafter.

[0065] The mannequin **30** is generally shown in FIG. **1** as having a torso **31**, or trunk, limbs **32**, a neck **34** and a head **35**. In order for the system **10** to provide a realistic simulation of cervical spine motions, the mannequin **30** may be similar to the human body in terms of weight, dimensions, flexibility, center of mass, and/or range of motion. While the system **10** focusses on simulating the cervical spine motions and providing data relative to neck movement, it is observed that torso **31** manipulations may affect the cervical spine stability. Therefore, by having the mannequin **30** emulating a human body in terms of weight, dimensions, flexibility, center of mass, and/or range of motion, transfer motions of the mannequin **30** may be realistic and therefore simulate adequately cervical spine motions. As part of sensors A, it is contemplated to provide various inertial sensors on or in the mannequin **30**, for instance to evaluate the forces of movement of the mannequin **30** when some specific transfer motions are done such as rotating the mannequin **30**, using the force evaluating module **20**D.

[0066] Referring to FIGS. **2** to **5**, a neck mechanism of the mannequin **30** is shown at **40**, while a skull is shown at **50**. The neck mechanism **40** interfaces the skull **50** to the torso **31**. According to an embodiment, the neck mechanism **40** and skull **50** are covered by a material, layers, or like cover emulating soft tissue, but the covering material is removed from FIGS. **2-5** to better illustrate the neck mechanism **40** and skull **50**.

[0067] The neck mechanism **40** is designed to simulate movements of the cervical spine, again in terms of weight, dimensions, flexibility/resistance, center of mass, and/or range of motion. According to an embodiment, the neck mechanism **40** allows movements of the skull **50** relative to the torso **31** about three or more axes, with four distinct axes shown in FIGS. **2-5**, although there may be fewer or more of these axes. The axes of the described embodiment are shown in FIG. **2**. According to one possible embodiment, there are two flexion axes shown as X1 and X2. The flexion axes X1 and X2 may generally be aligned with the lateral axis of the mannequin **30**. The flexion axes X1 and X2 may be generally parallel to one another. A rotation of the skull **50** relative to the torso **31** may be permissible about axis Z

(aligned with the cranial-caudal axis of the mannequin **30**), whereas a lateral flexion of the skull **50** relative to the torso **31** may be permissible by way of axis Y, generally aligned with the anterior-posterior axis of the mannequin **30**.

[0068] To allow such rotational movements, the neck mechanism **40** may therefore have three or more joint units, such as four distinct joint units **41** as in FIGS. **2-5**, also known as joint assemblies. The joint units **41** are serially interconnected. In an embodiment, an interconnection between one or more of sets of two adjacent joint units **41** forms a rotational joint of the type providing one rotational degree of freedom (DOF). In FIGS. **2** to **5**, the three lower joint units **41** are generally similar in configuration, but are oriented differently. For example, a bottommost one of the joint units **41** has its rotational axis aligned with axis Y. The second and third of the joint units **41** from the bottom are aligned respectively with axes X1 and X2. A top of the joint units, shown as **41'**, is aligned with the Z axis. Other alignment arrangements are possible as well. The top joint unit **41'** forms a rotational joint with the skull **50**, and provides a fourth DOF to the assembly of the neck mechanism **40** and skull **50**.

[0069] Reference is now made to FIG. **3**, in which the third one of the joints **41** from the bottom is shown in greater details. The components of the third joint **41** are also present in the two bottom joints **41**, and as such the two bottom joints **41** are not broken down to avoid redundancy in the text, and an excess of reference numerals in the figures. Each of the joint units **41** has a casing **42** and a base **43**. The casing **42** may have a pair of side walls **42**A, which serve a structural function in holding other parts of the casing **42** together. The side walls **42**A face each other and are spaced apart from one another. The side walls **42**A may each slidingly support an abutment **42**B. For this purpose, the side walls **42**A are shown as having vertical slots, by which the abutments **42**B may be slidingly attached for vertical movement, until a desired position is reached. Fasteners (not shown) may then be used to fix the abutments **42**B in position along the side walls **42**A. As detailed hereinafter, the abutments **42**B may serve to block movement of an adjacent joint unit **41/41'** and hence be a limit stop.

[0070] Journal walls **42**C are transversely oriented relative to the side walls **42**A and are connected to them, to concurrently define a cavity. In an embodiment, the side walls **42**A and the journal walls **42**C could be made of a single monolithic piece, such as a tube (e.g., square section tube). The journal walls **42**C each may have a bearing, to rotatingly support a shaft **42**D. Any appropriate type of bearing may be used, including ball bearings, roller bearings, journal bearing, low-friction sleeves, etc. A shaft interface **42**E is also mounted to the shaft **42**D. The shaft interface **42**E is used to connected the joint unit **41** with the adjacent joint unit **41/41'** as detailed hereinafter. A clamping collar **42**F may also be present to ensure that the shaft **42**D remains in its casing **42**. The walls **42**A and **42**C may be interconnected by any appropriate way (fasteners, welding, etc), but may also be monoblock or integrally formed. A sensor **42**G (part of the sensors A of FIG. **1**), is mounted to one of the walls of the casing **42**, and senses the rotation of the shaft **42**D. For example, the sensor **42**G is a rotary encoder, or any other type of sensor used to measure an angular displacement of the shaft **42**D. While the casing **42** is shown as a plurality of components, some of the components may be regrouped in monolithic pieces, etc.

[0071] The base **43** of the joint unit **41** supports the casing **42**. Hence, the base **43** has a plate **43**A upon which are connected the side walls **42**A and the journal walls **42**C. A plunger **43**B projects from the plate **43**A. In the illustrated embodiment, the plunger **43**B projects downwardly relative to the Z axis, but the reverse arrangement is also contemplated, with the joint units **41** oriented such that the plungers **43**B project upwardly relative to the Z axis.

[0072] In FIG. **3**, the plunger **43**B of the exploded joint unit **41** is only partially visible as it projects into the cavity of the lower joint unit **41**. However, the plunger **43**B of the upper joint unit **41'** is visible, and has the same configuration as the one that is hidden. The plunger **43**B has a head featuring a bore **43**C, through which the shaft **42**D may pass. The shaft interface **42**E is fixed to the head of the plunger **43**B, and as the shaft **42**D passes through the shaft interface **42**D, a rotational joint is formed between the plunger **43**B of the upper joint unit **41/41'** and the casing **42** of the lower joint unit **41**. Accordingly, the exploded joint unit **41** of FIG. **3** allows rotational movement of the upper plunger **43**B relative to the casing **42**, along the second flexion axis X2. As for the bottom most one of the joint units **41**, its plunger **43**B visible in FIG. **2** may be anchored to the torso **31**, although it may also provide a rotation DOF. Similar arrangements are reproduced between the first and second joint units **41** to allow lateral flexion about axis Y, and between the second and third joint units **41** to allow flexion about axis X1.

[0073] Referring to FIG. **4**, the top joint unit **41'** is shown in an exploded view. The joint unit **41'** has numerous components in common with the joint units **41** described above with reference to FIG. **3**, whereby like reference numerals are indicative of like components. It is however observed that the shaft **42**D is vertical in the top joint unit **41'**, whereby the side walls **42**A and the journal walls **42**C are oriented differently than in the other joint units **41**. The shaft **42**D and shaft interface **42**E of the joint unit **41'** are not connected to the plunger of another one of the joint units **41**, but are instead connected to a plunger **45**A centrally located in a disk **45** or disk ring. The plunger **45**A has a similar configuration as the plungers **43**B, but it is part of the disk **45**. In an embodiment, the plunger **45**A is an integral monoblock part of the disk **45**. Accordingly, the disk **45** may rotate about the vertical axis Z by way of its connection to the shaft **42**D of the joint unit **41'**. A pair of tabs **45**B may be on the underside of the disk **45**. The tabs **45**B may be used as attachments for a head shell that would be positioned onto the skull **50**. The disk **45** is the interface between the neck mechanism **40** and the skull **50**. The skull **50** is therefore anchored to the disk **45** and rotates with it. As an alternative to a disk **45**, a frame could be used.

[0074] Referring to FIG. **5**, the skull **50** may be composed of various plates **51** and brackets **52**, with a central plate **51**A mimicking the arcuate shape of a human skull. As the skull **50** may be the support for layers of material imitating soft tissue, the central plate **51**A may define the rounded skull shape, whereas the other plates **51** and brackets **52** give the skull **50** its appropriate diameters and emulate the volume of a skull, as well as the weight and/or center of mass. Moreover, the plates **51** and brackets **52** provide some mass to the skull **50** and therefore allow it to be representative of a real skull. According to one embodiment, the arrangement of plates **51** and brackets **52** aims to have the center of mass of the skull **50** coincide with the center of mass of a human

skull. It is therefore contemplated to use a material such as metal for many of the components of the neck mechanism **40** and skull **50**. Also, tendons **53** may be used to emulate the elasticity of soft tissue. For example, the tendons **53** may be elastic bands or cables. Four of the tendons **53** are shown, to give some balance to the assembly. Although not shown, the tendons **53** are attached to anchor points on the torso **31**. The elasticity and/or tension of the tendons **53** are also variables that may affect the realism of the mannequin **30**.

[0075] Although a given configuration of joint unit **41**/**41**' is detailed above, numerous other configurations are considered. For example, instead of using numerous joint units **41**, it is contemplated to provide a joint with multiple rotational DOFs (such as a universal joint or a spherical joint). Moreover, the joint units **41** may have a different configuration, for instance by being simpler hinges made of a bracket and shaft. Sensors of different nature may be used, such as gyroscope, accelerometers, instead of magnetic rotary encoders or optical rotary encoders. Numerous other configurations apply. In contrast to a universal joint, the rotational axes Y, X**1** and X**2** do not share a common point of intersection. The spacing apart of the lateral (X**1**, X**2**) and anterior-posterior (Y) rotational axes in the neck mechanism **40** may be a realistic emulation of a human spine.

[0076] Referring to FIG. **7**, a mannequin skeleton is generally shown at **70**. The mannequin skeleton **70** may or may not be part of the system **10**. The neck mechanism **40** and skull **50** may be used with the mannequin skeleton **70**, to emulate the inertia and elasticity of an unanimated human body, with the inertial of unanimated limbs. The skeleton **70** generally has a torso portion **80**, and a lower body portion **90**. In the illustrated embodiment, the demarcation between the torso portion **80** and a lower body portion **90** is around a waist line of the skeleton **70**. According to an embodiment, the skeleton **70** may be overmolded with a resilient material, such as a rubber or silicone, that will provide a soft tissue appearance and flexibility to the skeleton **70**. When the skeleton **70** is overmolded, it may have the appearance of the mannequin **30** of FIG. **1**.

[0077] Referring to FIG. **8**, the torso portion **80** has various components, generally described as a central member **81**, shoulder blades **82**, resilient joints **83** and arms **84**. The central member **81** is the spinal component of the torso portion **80**. In the illustrated embodiment, the central member **81** has a connector **81**A for interface with the neck mechanism **40** (FIG. **2**), for instance by way of fixed connection (no relative movement once locked to one another). As a possibility, the connection of the neck mechanism **40** with the torso portion **80** may be by way of a rotational joint, providing one, two or three rotational degrees of freedom (e.g., the connector **81**A forming with the neck mechanism **40** a spherical joint or universal joint). The connector **81**A is at an end of spinal member **81**B, that extends along the cranial-caudal axis of the torso portion **80**. The spinal member **81**B may flare downwardly (though other contours are also considered), and join an abdominal member **81**C. The spinal member **81**B and abdominal member **81**C have a width proportional to a rib cage. The abdominal member **81**C may form a cavity to receive the electronic components of the system **10**. A lower spinal member **81**D projects downwardly from the abdominal member **81**C, for connection to a lower body of the mannequin. The above configuration for the central member **81** is one of numerous possible configurations. The configuration described above is rigid to be representative of the rigidity of the rib cage portion of a human torso. Shoulder blades **82** are connected to the central member **81** by way of resilient joints **83**, to emulate the soft tissue flexibility in the shoulder region of a human body. This soft tissue flexibility in the human shoulder region poses a challenge in roll-over of the body, as cervical spine stability can be affected by this looseness in the torso. The arrangement of central member **81**, shoulder blades **82** and resilient joints **83** replicates the human torso in the roll-over manipulations.

[0078] Each of the shoulder blade **82** may have a polygonal body, though other shapes are contemplated as well, for instance made out of a plate. Connection strips **82**A are provided along edges of the shoulder blades **82**, for connection with the resilient joints **83**. Three resilient joints **83** are present, with an elongated resilient joint **83** being between the spinal member **81**B and each of the two shoulder blades **82**. The resilient joints **83** may be made of a rubbery material, such as a rubber or polymer. For example, a silicone may be used as material for the resilient joints **83**, with a suitable density to simulate the shoulder soft tissue (tendons, muscles). According to an embodiment, instead of the three discrete resilient joints **83**, the overmolding of rubbery material joins the shoulder blades **82** with the central member **81**, while emulating soft tissue flexibility present in a human shoulder area by the absence of a rigid connection between the shoulder blades **82** and the central member **81**. Although not shown, elastic bands may also be provided to limit the free movement of the shoulder blades **82** relative to the central member **81**. Hooking members **82**B are on each shoulder blade **82**, for attachment of the tendons **53** (FIG. **2**). The hooking members **82**B may have any appropriate shape or configuration, including holes, slots, projecting connectors, etc. A glenoid member **82**C may be provided in each of the shoulder blades **82**, for connection of the arms **84** to the shoulder blades **82**. The glenoid member **82**C may be any appropriate bracket for interfacing the arms **84** to the plates or bodies of the shoulder blades **82**. As a possibility, the glenoid member **82**C may be a rotational joint, providing one, two or three rotational degrees of freedom, to emulate a human shoulder joint. It is also observed that numerous holes are distributed over the torso portion, such as in the spinal member **81**B and the shoulder blades **82**. These numerous holes may be used for the connection of weights to adjust a position of a center of mass of the torso portion **80**.

[0079] The arms **84** are illustrated as having a sequence of joints and links to reproduce the shoulder joint, upper arm, elbow and lower arm of a human body. According to an embodiment, each arm **84** has a sequence of a first rotational joint **84**A, a second rotational joint **84**B, an upper arm member **84**C with movable weights **84**D, an elbow rotational joint **84**E, a lower arm member **84**F and a wrist rotational joint **84**G. This is one of numerous configurations. For example, instead of rotational joints, the arms **84** may have the members **84**C and **84**F, as well as a hand member (not shown, part of the rotational joint **84**G), be interconnected by the resilient rubbery material overmolded onto the skeleton **70**.

[0080] The combination of first rotational joint **84**A and second rotational joint **84**B connected to the glenoid members **82**C of the shoulder blades **82** concurrently form two rotational DOFs between the shoulder blades **82** and the upper arm member **84**C, similar to the motion range of a

human shoulder joint. As an another example, a spherical joint is provided instead of the arrangement of the two joints **84**A and **84**B. The first rotational joints **84**A have their rotational axis generally parallel to the X axis of the coordinate system, while the second rotational joints **84**B have their rotational axis transverse relative to the rotational axis of first rotational joints **84**A. The upper arm members **84**C and/or the lower arm members **84**F may be threaded rods upon which the weights **84**D may be moved to adjust the position of the center of mass of the members **84**C and **84**F. The elbow rotational joints **84**E and wrist rotational joints **84**G have their axes of rotation generally transverse to a longitudinal axis of the members they are connected to, so as to reproduce elbow and wrist movements. More or less rotational joints may be present as well, such that the arms **84** have some flexibility at the shoulder, elbow and wrist joints.

[0081] Referring to FIG. **9**, the lower body portion **90** has a pelvis member **91**, and a pair of legs **94** projecting from the pelvis member **91**. It is observed that the pelvis member **91** is not rigidly connected to the central member **81**, with a gap being defined between the lower spinal member **81**D and the pelvis member **91**. According to an embodiment, a resilient joint (e.g., silicone) or the resilient rubbery material overmolded over the skeleton **70** flexibly join the torso portion **80** to the lower body portion **90**. The pelvis member **91** may be an inverted U-shaped bracket to which the legs **94** are connected. The legs **94** may have a configuration similar to the arms **84**. The legs **94** are illustrated as having a sequence of joints and links to reproduce the high joint, thigh, knee and lower leg of a human body. According to an embodiment, each leg **94** has a sequence of a first rotational joint **94**A, a second rotational joint **94**B, a thigh member **94**C, movable weights **94**D, a knee rotational joint **94**E, a lower leg member **94**F and an ankle rotational joint **84**G, among possible configurations. Again, instead of rotational joints, the legs **94** may have the members **94**C and **94**F, as well as a foot member (not shown, part of the rotational joint **94**G), be interconnected by the resilient rubbery material overmolded onto the skeleton **70**.

[0082] The combination of first rotational joint **94**A and second rotational joint **94**B connected to the pelvis member **91** concurrently form two rotational DOFs between the pelvis member **91** and the thigh members **94**C, similar to the motion range of a hip joint. As an another example, a spherical joint is provided instead of the arrangement of the two joints **94**A and **94**B. The first rotational joints **94**A have their rotational axis generally parallel to the X axis of the coordinate system, while the second rotational joints **94**B have their rotational axis transverse relative to the rotational axis of first rotational joints **94**A. The thigh members **94**C and/or the lower leg members **94**F may be threaded rods upon which the weights **94**D may be moved to adjust the position of the center of mass of the members **94**C and **94**F. The knee rotational joints **94**E and ankle rotational joints **94**G have their axes of rotation generally transverse to a longitudinal axis of the members they are connected to, so as to reproduce knee and ankle movements. More or less rotational joints may be present as well, such that the legs **94** have some flexibility at the hip, knee and ankle joints.

[0083] Referring to FIGS. **10** and **11**, an airway simulator apparatus **100** is shown as part of the mannequin **30**, used in collaboration with the torso **31**, the neck mechanism **40** and the skull **50**. Although not shown, the mannequin **30** featuring the airway simulator apparatus **100** may also have a mannequin skeleton **70**. Moreover, as observed from FIGS. **10** and **11**, the mannequin **30** may have a soft tissue membrane emulating skin, with the airway simulator apparatus **100** being accessible through a mouth of the mannequin **30**, or through nostrils of the mannequin **30**, the mouth and the nostrils being defined in the skin of the mannequin **30**. The airway simulator apparatus **100** emulates anatomical airways, with at least the following components: a mouthpiece **101**, a throat tube **102**, a pharyngeal tube **103**, nose tube(s) **104**, oesophageal tube **105**, stomach **106**, tracheal tube(s) **107** and lungs **108**.

[0084] The mouthpiece **101** opens to the month in the skin of the mannequin **30**. The mouthpiece **101** may taper toward the throat tube **102**. The throat tube **102** may then connect with the pharyngeal tube **103**. In an embodiment, the pharyngeal tube **103** has a larger diameter than the throat tube **102**. Nose tube(s) **104** also merge with the pharyngeal tube **103**. As shown in FIG. **10**, there may be a part of nose tubes **104**, extending all the way to the pharyngeal tube **103**. Alternatively, the nose tubes **104** may merge before connected to the pharyngeal tube **103**. In the shown arrangement, the throat tube **102** and the pharyngeal tube **103** all merge with a common top end of the pharyngeal tube **103**, for instance via an interface.

[0085] The pharyngeal tube **103** extends toward the torso **31**. Though it bears the moniker “pharyngeal”, the pharyngeal tube **103** may be longer than an anatomical pharynx. The pharyngeal tube **103** may then diverge into the oesophageal tube **105** connected to the stomach **106**, and into the tracheal tubes **107** connected to the lungs **108**. The stomach **106** and the lungs **108** may be expansible balloons or like vessel of elastic material, which can expand when pressurized. In an embodiment, the airway simulator apparatus **100** is generally airtight, such that air blown into the airway simulator apparatus **100** via the mouthpiece **101** or the nose tubes **104** may result in the inflation of the lungs **108**, if not also of the stomach **106**. Accordingly, maneuvers such as mouth-to-mouth resuscitation may be practiced with the mannequin **30** featuring the airway simulator apparatus **100**. The airway simulator apparatus **100** is shown as having both a stomach **106** and lungs **108**, but the airway simulator apparatus **100** may be limited to either one of the stomach **106** and lungs **108**. The various tubes of the airway simulator apparatus **100** may be of a polymer that has a level of elasticity similar to that of human airways. The tubes of the airway simulator apparatus **100** may consequently be flexible tubing. The material used for the stomach **106** and/or the lungs **108** may be different, due to capacity of these human cavities to inflate. As the airway simulator apparatus **100** is under the skin of the mannequin **30**, the arrangement emulates the elasticity of human tissue on the human airways. In an embodiment, the airway simulator apparatus **100** is connected to the skull **50**, extends longitudinally along the neck mechanism **40**. More specifically, the nose tubes **104** may be connected to the central plate **51**A of the skull **50**, among one possibility.

[0086] As direct laryngoscopy and tracheal intubation are necessary skills in airway management, and thus the airway simulator apparatus **100** may contribute to the training for airway management. Glottis visualization may be effect with the airway simulator apparatus **100**, as it is necessary to perform successfully direct laryngoscopy and intubation. This requires proper positioning of head and neck to visu-

alize the glottis and easily access the tracheal tube through the glottic opening, whereby tracking of the orientation of the neck mechanism **40** and skull **50** may be used in conjunction with the airway simulator apparatus **100**. It is known that an optimal position of the patient's head and neck at the time of laryngoscopy and intubation may have an impact on the outcome, and one known position is referred to as "sniffing position" (SP). The system **10** may therefore be programmed to measure and/or give feedback about what constitutes the range of angles of the "sniffing position" for the neck mechanism **40** and skull **50**, and monitor the rotations about X**1**, X**2**, Y and Z during simulated direct laryngoscopy and/or tracheal intubation.

[0087] Accordingly, the airway simulator apparatus **100** defines a realistic airway anatomy and landmarks including pharynx, larynx, epiglottis, arytenoids, false cords, true vocal cords, trachea, lungs, esophagus structures, with some or all of these imbedded in a flexible tubes of the airway simulator apparatus **100**. As the airway simulator apparatus **100** is attached to the mechanical structure of the mannequin **30** (e.g., skull **50** as above, or otherwise neck mechanism **40**), the airway simulator apparatus **100** changes position with the position of the skull **50** (neck flexion and head extension). The inertia of the skull **50** in combination with the degree of motions motion offered by the neck mechanism **40** allows a more realistic and sensitive positioning of the skull **50** to achieve the sniffing position for the airway simulator apparatus **100**. The sensors in the neck mechanism **40** allow the measurements and feedback of the alignment of sniffing position during the intubation and subsequent superfluous head motions in all of the anatomical planes during the intubation maneuvers. The system **10** therefore proposes a quantitative approach to measure the efficacy of the c-spine stabilization and provide objective feedback during training. The proposed quantitative approach has the potential to be used for personalized feedback during training sessions.

[0088] Using the readings from the sensors A, the performance assessor module **20**C may perform a characterization of the quality of transfer maneuvers based on the analysis of the temporal and spatial gap between the actual manoeuvre and the ideal representation of the motion, for example during a log-roll. In a perfect log-roll, both head and trunk segments move at the same time (i.e. temporal synchronicity), following concordant paths or "en-bloc" (i.e. spatial synchronicity). The global change in orientation for each segment should therefore be the same throughout the maneuver, and the sensors A can therefore be used to detect variations. Unwanted motion (e.g. head dropped in extension during the roll) will be captured in the global change in orientation experienced by the skull **50**.

[0089] Temporal synchronization refers to the ability of the rescuers to move the trunk and the head at the same time. Poor communication between the rescuers or difficulty initiating and maintaining smooth motion of the trunk during the roll and push phases (e.g. due to a lack of strength with a large SP) may cause a delay between the motion of the head and the motion of the trunk, specifically at the initiation of a specific phase of a motion. Such delay will therefore be investigated during the initiation of the two phases requiring most of the motion (i.e. roll and push).

[0090] Spatial synchronicity refers to the idea that both segments move along a proportional arc of circle during both the roll and the push phases, represented by that desired line of identity on a 2D motion graph as in FIG. **6**. Potential spatial quality indicators may therefore be monitored by the performance assessor module **20**C based on the assumptions regarding ideal log-roll and its representation using a 2D motion graph approach). As such, the deviation from the desired line of identity during the roll and the push phases is investigated as a potential indicator, using the roll and the push best-fit line determined by a least-square approach. An efficient log-roll also assumes that the lead rescuer and assistant(s) have sufficient control over the motion. Perfect control will allow the rescuers to follow the same path throughout the roll and the push phases. Hence, it is hypothesized that the larger the spread between the two curves on the motion phase-plane, the worse the control and hence the result. One way of capturing that "spread" is by calculating the area between the curves. Hence, that area between the curves will be investigated as a possible indicator of acceptable and unacceptable log-rolls. Table 1 below provides performance and quality indicators for log-roll, using data measurements as in FIG. **6**.

TABLE 1

Performance and Quality Indicators for Log-Roll

CATEGORY	VARIABLE	EQUATION	DESCRIPTION
Performance measure	$ROMrel_{peak}$	Max(ROMrel)	Peak change in global orientation of the head relative to the trunk.
Temporal Quality Indicators	$Delay_{roll\_ini}$	⑦	Delay at roll Initiation
	$Delay_{roll\_end}$	⑦	Delay at Roll termination
Spatial Quality Indicators	$Slope_{Roll}$	⑦	Difference between the slope of the best-fit line of the Roll curve and the ideal line of identify.
	$Slope_{Push}$	⑦	Difference between the slope of the best-fit line of the Push curve and the ideal line of identity.
	$ABC_{Roll\text{-}Push}$	⑦	Area contained between the curves from the Roll and the Push phases.

⑦ indicates text missing or illegible when filed

1. A neck mechanism for a mannequin comprising:

at least three joint units serially connected to provide joints for at least three rotational degrees of freedom (DOF), with a rotational axis of a first DOF configured to be aligned with a lateral axis of the mannequin, a rotational axis of a second DOF configured to be aligned with an anterior-posterior axis of the mannequin, and a rotational axis of a third DOF configured to be aligned with a cranial-caudal axis of the mannequin,

wherein a bottom one of the at least three joint units is adapted to be connected to a torso of the mannequin, and a top one of the at least three joint units is adapted to be connected to a skull.

2. The neck mechanism according to claim **1**, wherein the bottom one of the at least three joint units is adapted to be connected to the torso of the mannequin with the second DOF.

3. The neck mechanism according to claim **1**, wherein the top one of the at least three joint units is adapted to be connected to a skull by the third DOF.

4. The neck mechanism according to claim **1**, comprising four joint units, providing concurrently four rotational DOFs, wherein a rotational axis of a fourth DOF is configured to be aligned with the lateral axis of the mannequin.

5. The neck mechanism according to claim **4**, wherein the rotational axes of the first DOF and of the fourth DOF are parallel and spaced apart to another.

6. The neck mechanism according to claim **5**, wherein the rotational axes of the first DOF and of the fourth DOF are parallel and spaced apart relative to the rotational axis of the second DOF.

7. The neck mechanism according to claim **1**, comprising a rotary motion sensor at each said joint unit.

8. A mannequin comprising:

the neck mechanism according to claim **1**;

a skull connected to the top one of the at least three joint units; and

a trunk connected to the bottom one of the at least three joint units.

9. The mannequin according to claim **8**, wherein the trunk has a plurality of metal plates emulating a volume and/or a weight of an anatomical skull.

10. The mannequin according to claim **8**, wherein the skull has a disk ring connected to a shaft of the top one of the at least three joint units.

11. The mannequin according to claim **8**, comprising elastics connecting the skull to the trunk.

12. The mannequin according to claim **8**, further comprising an airway simulator apparatus having at least one tube defining at least one opening at a level of a face of the mannequin, and being in fluid communication with at least one expandable balloon in the trunk.

13. The mannequin according to claim **12**, wherein the airway simulator apparatus includes a mouthpiece connected to the at least one tube at the at least one opening.

14. The mannequin according to claim **12**, wherein the at least one tube includes at least one nose tube having an opening defining a nostril.

15. The mannequin according to claim **12**, wherein the at least one tube diverges into at least two tracheal tubes, with one said expandable balloon at an end of each said tracheal tube.

16. The mannequin according to claim **15**, wherein the at least one tube diverges into an oesophageal tube, with one said expandable balloon at an end of the oesophageal tube.

17. The mannequin according to claim **12**, wherein the at least one tube is flexible tubing.

18. The mannequin according to claim **12**, wherein the at least one tube is connected to the skull.

19. The mannequin according to claim **8**, wherein the trunk comprises an articulated skeleton.

20. The mannequin according to claim **8**, wherein the neck mechanism, the skull and/or the trunk are covered by a skin membrane.

21.-**41**. (canceled)

* * * * *
